# Deep learning-supported image quantification of epithelial cell shapes and its application to polycystic kidney disease

**DOI:** 10.1371/journal.pcbi.1014614

**Published:** 2026-08-03

**Authors:** Johannes Jahn, Alexis Hofherr, Clara Consoli, Berenike Fajen, Rebekka Goll, Greta Theresa Liedtke, Adrian Boehm, Friederike Selbach, Paul Christoph Zeisler, Vanessa Weichselberger, Anne-Kathrin Classen, Lukas Westermann, Tilman Busch, Michael Köttgen

**Affiliations:** 1 Department of Medicine IV-Nephrology and Primary Care, Faculty of Medicine and Medical Center, University of Freiburg, Freiburg, Germany; 2 Department of Radiology, Medical Center – University of Freiburg, Faculty of Medicine – University of Freiburg, Freiburg, Germany; 3 Hilde-Mangold-Haus, University of Freiburg, Freiburg, Germany; 4 Faculty of Biology, University of Freiburg, Freiburg, Germany; 5 CIBSS Centre for Integrative Biological Signalling Studies, University of Freiburg, Freiburg, Germany; 6 Spemann Graduate School of Biology and Medicine (SGBM), University of Freiburg, Freiburg, Germany; University of Zurich Faculty of Mathematics and Science: Universitat Zurich Mathematisch-Naturwissenschaftliche Fakultat, SWITZERLAND

## Abstract

Cell shape is a fundamental determinant of tissue architecture and organ function. In epithelial tissues, cytoskeletal organization and apical junctions regulate cell geometry, shaping functional tissue units. Disruption of these mechanisms is associated with diseases such as autosomal dominant polycystic kidney disease (ADPKD), in which epithelial organization is altered leading to cyst formation. Quantitative analysis of epithelial morphology can provide mechanistic insight, but existing approaches are often manual, low-throughput, and difficult to standardize. Here, we present a fully automated, deep learning-supported image analysis workflow for quantifying epithelial morphology in immunofluorescence images of zonula occludens protein 1 (ZO-1)-stained monolayers. Using a U-Net-based segmentation approach designed to mitigate out-of-focus regions, we extract standard cell shape features together with readouts tailored to the phenotype under study, including the R-index for junctional meandering and a border-based proxy for intercellular force transmission at shared cell-cell interfaces. We apply this workflow to genetically modified Madin-Darby canine kidney (MDCK) cell models of ADPKD and show that it captures genotype-associated differences in junctional organization that are not fully described by conventional shape descriptors alone. The workflow enables standardized, high-throughput phenotyping across large image datasets, reduces observer dependence, and supports analysis of mixed-cell experiments with genotype-resolved shared-border behavior. Together, these results establish a scalable framework for assay-specific quantification of epithelial morphology and junctional organization in defined experimental systems.

## Author summary

The shape of epithelial cells is critical for organ function. In the kidney, properly shaped epithelial cells assemble into tubules ensuring efficient waste excretion as well as body electrolyte and water balance. Disruption of cell shape regulation can lead to diseases such as autosomal dominant polycystic kidney disease (ADPKD), characterized by cyst formation and displacement of normal kidney tissue. However, measuring changes in cell shape has often relied on manual inspection of microscope images, which is slow, subjective, and difficult to scale. In this study, we developed an automated image-analysis workflow that measures epithelial cell shape and the organization of cell borders from fluorescence microscopy images. We applied this approach to kidney epithelial cells carrying disease-related genetic changes and found clear differences in how the cells and their borders were organized. Our method makes it possible to analyze large image datasets in a standardized and reproducible way. We hope this approach will help researchers study how epithelial tissues are altered in disease and provide a useful tool for comparing cellular phenotypes across experiments.

## Introduction

Epithelial cells are the fundamental building blocks of many organs, and their ability to adopt specific shapes underlies critical morphogenetic processes during development [1]. Cell shape changes, including apical constriction, drive tissue folding, tubulogenesis, and lumen formation [2]. These processes are powered by the actomyosin cytoskeleton and coordinated through cell-cell junctions, particularly adherens and tight junctions [3]. *In vivo*, pulsed actomyosin contractions and junction-length fluctuations remodel epithelial cell outlines during morphogenesis, showing that irregular or dynamic cell borders are biologically meaningful readouts of cortical tension and junctional remodeling rather than passive by-products of tissue organization [4,5].

Tight junctions are particularly informative in this context because they are mechanically integrated with the apical cortex through zonula occludens (ZO) proteins, cingulin, actin, and non-muscle myosin [3,6–8]. Accordingly, tight-junction configuration can serve as a proxy for changes in cortical mechanics and junction-cytoskeleton coupling. Border-associated cytoskeletal traction provides a complementary readout of intercellular force transmission [9]. Perturbations of ZO-1, cingulin-dependent myosin tethering, or actin isoform balance alter tight-junction tortuosity or zig-zag morphology and affect epithelial morphogenesis in various Madin-Darby canine kidney (MDCK) systems [7,10,11]. This conceptual relationship between apical contractility, junction-cytoskeleton coupling, and junctional border meandering is summarized in Fig 1.

**Fig 1:**
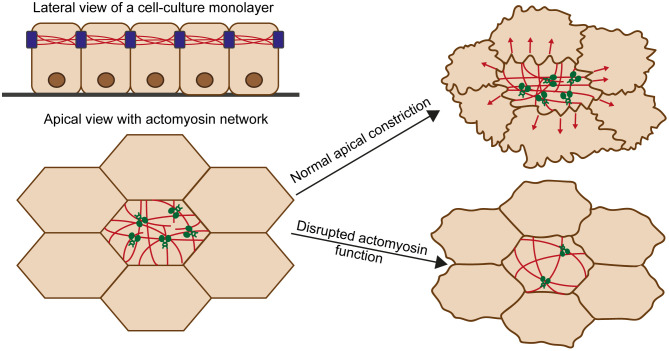
Relationship between apical constriction and junctional border meandering in epithelial monolayers. Upper left: Schematic lateral view of a confluent epithelial cell-culture monolayer. Lower left: Apical view illustrating a junction-associated actin (red) and myosin (green) network. Right panels: During normal apical constriction (upper right), contractile activity at the apical cortex and its coupling to cell-cell junctions is associated with tortuous/meandering cell borders. When junction-cytoskeleton coupling and/or actomyosin contractility is perturbed (lower right), borders adopt smoother, less tortuous configurations, consistent with reduced junctional remodeling. Conceptual schematic inspired by previous models of apical constriction [2].

At the organ level, the geometry of epithelial tissues directly contributes to organ shape and function. This is particularly evident in the kidney, where epithelial organization is essential for the formation and maintenance of functional tubular architecture [12]. Recent work further indicates that epithelial tension and cell shape changes contribute directly to morphogenesis of the developing kidney epithelium [13,14]. Disruption of the cellular mechanisms controlling epithelial morphology can lead to severe developmental defects and diseases, including Autosomal Dominant Polycystic Kidney Disease (ADPKD), a monogenic disorder caused by mutations in *PKD1* and *PKD2* [15,16]. These genes encode proteins involved in signal transduction at the primary cilium, and their dysfunction leads to altered cytoskeletal regulation, dedifferentiation, and cyst formation [8,17–22].

Despite the biological relevance of these phenotypes, quantitative analysis of epithelial morphology remains a bottleneck. Previous studies have provided mechanistic insight by manually quantifying tight-junction tortuosity, ruffling, or zig-zag morphology, and these measurements have helped link junctional architecture to cytoskeleton organization and function [10,23,24]. However, such analyses are typically time-consuming, user-dependent, and usually restricted to relatively small numbers of manually selected bicellular junction segments rather than entire monolayers, which limits scalability and reproducibility. Quantifying fine irregularities of tight-junction contours and border meandering across large and heterogeneous image sets is therefore still challenging.

Deep learning-based segmentation has greatly advanced biomedical image analysis [25–27] and has been incorporated into general image-analysis tools, including Cellpose and Tissue Analyzer, thereby advancing cell segmentation and epithelial morphometry [28,29]. In addition, dedicated tools such as TISMorph, Junction Mapper and Polarity-JaM further provide morphometric and junction-related descriptors [30–32]. However, no single out-of-the-box workflow combines robust segmentation with exclusion of out-of-focus regions, quantification of tight-junction meandering, and in particular genotype-resolved analysis of border deflection as a proxy for traction forces at shared cell-cell interfaces. This highlights the need for a scalable workflow tailored to quantifying these junctional phenotypes.

Here, we present a deep learning-supported workflow for automated analysis of epithelial morphology in ZO-1-stained monolayers. Using genetically modified MDCK models of ADPKD and related perturbations of junctional or cytoskeletal regulation, we combine robust segmentation with quantitative descriptors of tight-junction configuration and a border-based proxy for cytoskeletal traction. The workflow enables standardized high-throughput phenotyping across large image datasets and is compatible with mixed-cell experiments, in which heterotypic cell-cell borders can be analyzed within the same epithelial field. Overall, this work establishes a scalable workflow for automated phenotyping of epithelial morphology across epithelial model systems.

## Results

### Tight junction meandering as a morphological phenotype

To characterize junctional morphology in polarized kidney epithelial cells, we imaged the tight-junction-associated protein ZO-1 in MDCK monolayers. Wild-type (WT) and *PKD2*^*-/-*^ cells provide a genetically defined comparison between normal epithelial organization and an ADPKD-related perturbation of epithelial architecture. Given the established role of polycystins in epithelial organization, we asked whether loss of *PKD2* is associated with an altered tight-junction morphology that can be detected in ZO-1-stained monolayers. Immunofluorescence revealed distinct tight junction patterns in WT and *PKD2*^*-/-*^ MDCK cells. WT cells showed heterogeneous, meandering tight junctions with a pronounced riffled pattern, whereas *PKD2*^*-/-*^ cells displayed smoother, more uniform junctions (Fig 2). Re-expression of *PKD2* partially rescued this phenotype by restoring the characteristic riffled pattern (S1D Fig), and *PKD1*^*-/-*^ cells showed a similar reduction in junctional meandering (S1B Fig). Although genotype-associated differences were clearly visible in some fields, their magnitude varied across images (S2 Fig), limiting reliable genotype discrimination by blinded visual inspection and motivating quantitative analysis to measure and compare junctional meandering objectively.

**Fig 2:**
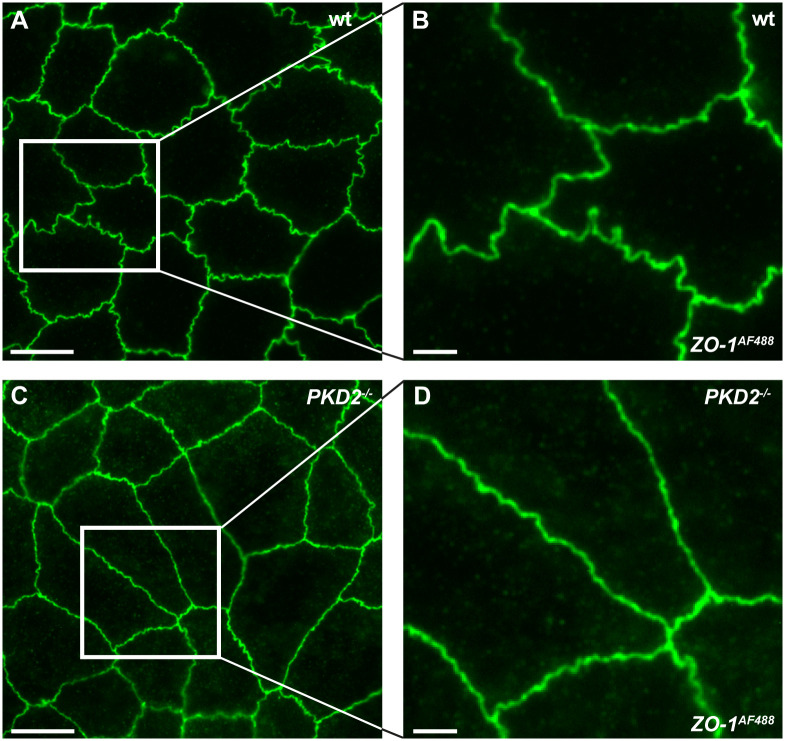
Tight junction meandering as a morphological parameter. Representative immunofluorescence images of MDCK WT and *PKD2*^*–/–*^ cells. Cells were stained with an α-Zonula occludens 1 primary antibody and an Alexa Fluor 488-conjugated secondary antibody. **(A)** WT cells show prominent membrane riffles, characterized by indentations and a meandering junctional pattern. **(B)** Enlarged view of panel **(A)**. **(C)** *PKD2*^*–/–*^ cells exhibit smooth cell borders with minimal ruffling lacking major indentations. **(D)** Enlarged view of panel **(C)**. Scale bars: 10 μm **(A, C)**; 2.5 μm **(B, D)**. MDCK = Madin-Darby Canine Kidney; *PKD2* = Polycystic kidney disease gene 2; WT = wild-type, ZO-1 = Zonula occludens protein 1.

### Benchmarking established tools for ZO-1 segmentation and meandering quantification

To assess whether established approaches are suitable for our ZO-1 imaging data and for tight-junction meandering quantification, we benchmarked CellProfiler, Cellpose-SAM, and Tissue Analyzer [28,29,33] on representative images and performed a qualitative comparision of the dedicated morphometric and junction-analysis tools TISMorph, Junction Mapper, and Polarity-JaM [30–32] (extensive Benchmarking in S1 Text). In brief, Cellpose-SAM and Tissue Analyzer produced strong deep learning-based segmentations with minimal setup, while CellProfiler enabled flexible classical threshold-based segmentation and feature extraction. However, the deep learning-based tools consistently segmented out-of-focus regions that are unsuitable for reliable meandering quantification in our assay, and Cellpose-SAM additionally smoothed fine junctional undulations. CellProfiler performance was less consistent across diverse image conditions, reflecting sensitivity to fixed thresholds and processing parameters. A qualitative comparison with TISMorph, Junction Mapper, and Polarity-JaM showed that existing tools provide comparable junctional descriptors, but are not directly tailored to our specific readout. None of the evaluated tools natively supported the combination of out-of-focus-aware segmentation, tight-junction phenotyping, and genotype-resolved shared-border analysis required for our screening workflow, mandating the development of a task-specific pipeline.

### Deep learning-based segmentation of ZO-1 images

We developed an end-to-end processing workflow that translates acquired images into quantifiable values, including tight junction morphology (Fig 3). To address the limitations of conventional threshold-based segmentation methods, we trained a deep learning segmentation system using a U-Net convolutional network to detect ZO-1-stained tight junctions while ignoring out-of-focus regions unsuitable for downstream meandering analysis (S3 Fig). On an independent test set (n = 3 images), evaluated after 10,000 training epochs, the model achieved an intersection over union (IoU) of 0.740, a Dice coefficient of 0.851, a precision (positive predictive value) of 0.773, and a recall (sensitivity) of 0.946. Deviations from the manual reference were typically small and localized to boundaries (usually ~1 pixel) and had only negligible effects on downstream morphometric analysis and the characteristic meandering pattern after skeletonizing. The comparatively moderate IoU is consistent with the segmentation target being a thin linear structure (~3–4 pixels wide), for which small boundary deviations disproportionately affect overlap-based metrics. In addition to metric-based evaluation, post hoc visual review by multiple researchers (n = 9) showed no systematic segmentation errors across varying background signals and artifacts, confirmed that the meandering pattern was sufficiently captured, and indicated adequate exclusion of out-of-focus regions. Because cell objects containing partially out-of-focus regions were excluded from downstream morphometric analysis, residual blur did not introduce a systematic bias in the reported morphology measurements, but instead reduced the number of analyzable cells. This reduction in cell count can be compensated by increasing the number of biological replicates, which can be generated with comparatively low experimental effort. Our setup processed approximately 1,000 images per hour based on our standard image format of 1388 × 1040 pixels, corresponding to approximately 0.015 mm² per image, enabling high-throughput analysis.

**Fig 3:**
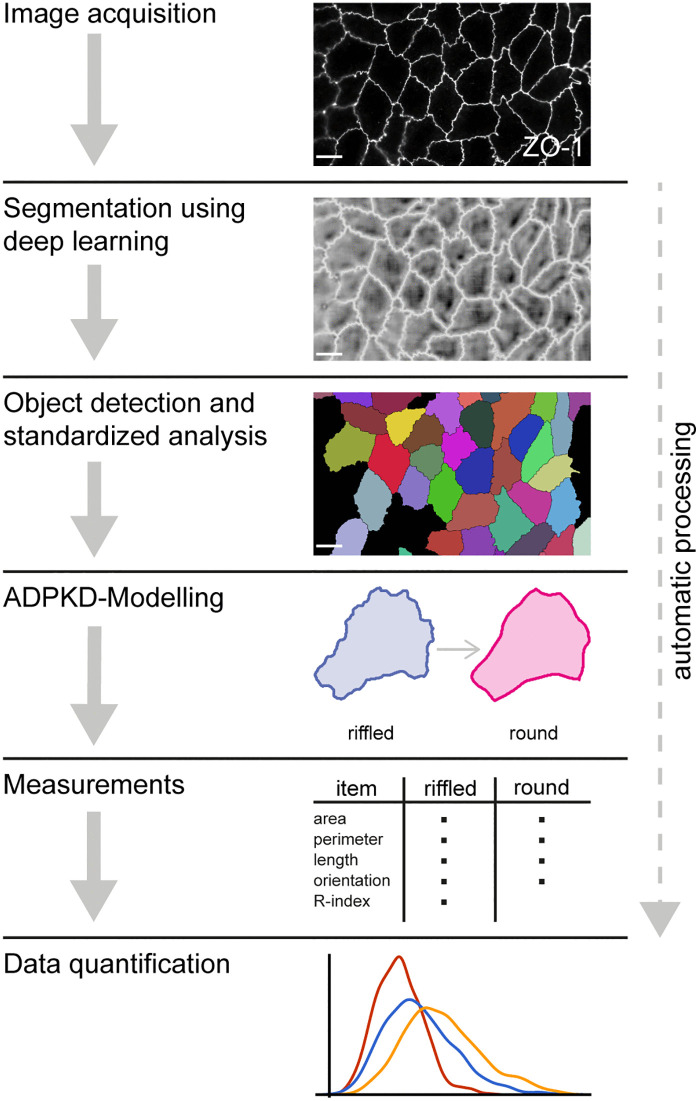
Schematic representation of the automated image analysis pipeline. Scale bars: 10 μm.

### Cell detection and standardized measurements

Further analysis steps were implemented in Mathematica 12 (Wolfram Research, Champaign, Illinois, USA). First, segmented images were pre-processed to remove background noise and eliminate incorrect segmentations. The filtered in-focus tight-junction network was then skeletonized to a single-pixel width to normalize variations in junction thickness observed across different genotypes and antibody combinations. Watershed algorithms were used to detect individual cell objects from the skeletonized image (S4 Fig), yielding measurements such as area, perimeter, and diameter for each object (Fig 4). Uniform quality-control filters were applied across all datasets and genotypes, excluding border-touching objects, objects lacking a closed/uninterrupted ZO-1 contour, objects with internal skeleton branch points (merged-cell candidates), and objects <25 µm² (below typical MDCK WT area: 165 ± 58 µm², n = 5,946). In a manually reviewed representative subset of 12 images, 734 of 1,173 cells in the field (62.6%) were excluded by these predefined criteria, including 456 border-touching cells (38.9%) and 278 cells excluded because of out-of-focus regions or segmentation errors (23.7%). After applying these fixed criteria, all remaining objects were included in downstream analyses without further dataset- or phenotype-specific selection. Manual inspection of a larger separate randomly selected subset yielded a false detection rate (under- or over-segmented objects, as well as fused or split detections) of 0.4% (24/5,477). All images were processed using the same scripted pipeline and quantified in physical units using the microscope pixel size, without further normalization to preserve true biological differences.

**Fig 4:**
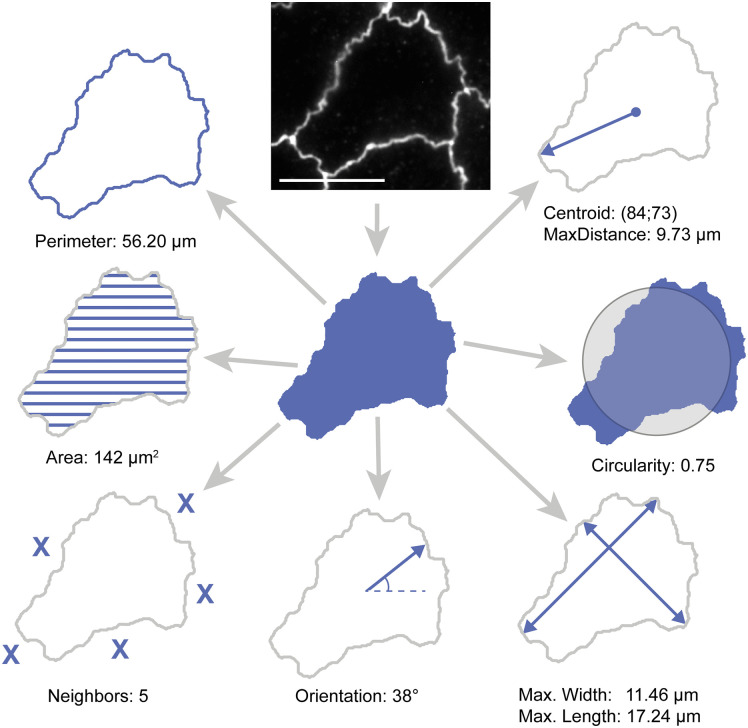
Visualization of potential measurement parameters extracted from cell objects. Measurements were obtained using the Mathematica function ComponentMeasurements [34]. Circularity was calculated as 2πr_eq_/p, where r_eq_ is the equivalent-disk radius and p is the polygonal perimeter of the segmented object; this corresponds to the square root of the conventional circularity measure (4πA/P²). Neighbor count was derived from the number of cell border segments connected to each cell after skeletonization of the ZO-1 network and separation at branch points. Scale bar: 10 µm. A = area; P = perimeter; ZO-1 = zonula occludens 1.

### Quantification of junctional meandering in ADPKD models

To better characterize the cellular ADPKD phenotype and tight junction architecture, we developed a model to quantify junctional meandering and riffled patterns. While conventional descriptors (e.g., circularity), can be easily calculated, they did not reliably reflect the degree of tight junction irregularity in blinded manual assessments. We therefore quantified perimeter excess relative to a reference contour obtained by smoothing the segmented tight junction network using a combination of blurring, erosion and edge filters (Figs 5A and S5). The strength of the smoothing algorithm was iteratively adjusted based on qualitative evaluation by five independent laboratory members to suppress high-frequency contour features while preserving overall cell size and orientation. Cells with pronounced riffles showed a larger deviation between the original and smoothed object perimeters, whereas less riffled junctions showed minimal deviation (Fig 5B).

**Fig 5:**
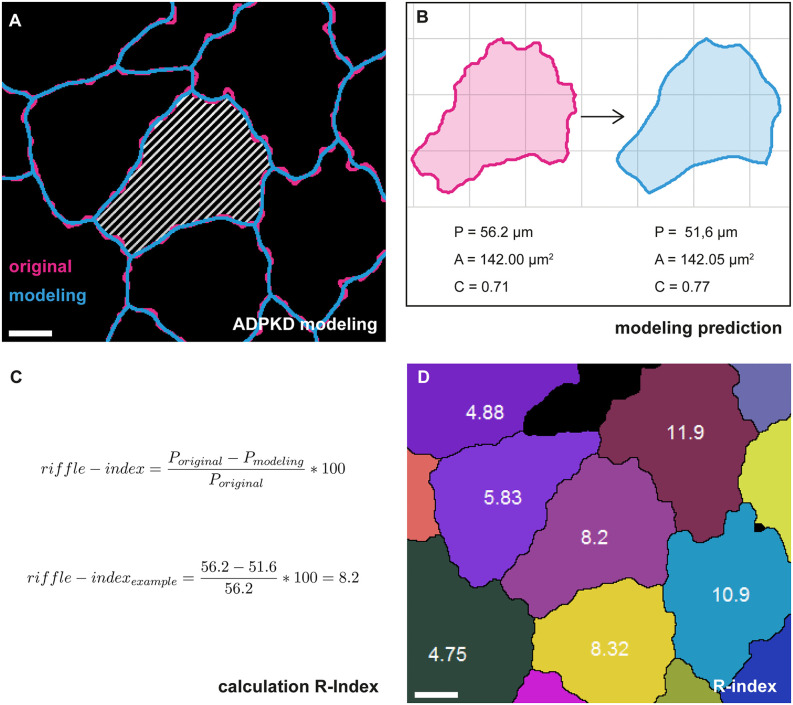
ADPKD modeling and computation of the R-index. **(A)** Overlay of the original segmentation (magenta) and the smoothed model (blue) of wild-type cells. Larger differences are observed when the original cell border is heterogeneous and displays pronounced ruffling. **(B)** Comparison of perimeter, area, and circularity between the original and smoothed cell objects. **(C)** Illustration of R-index computation based on perimeter deviation. **(D)** Representative R-index values derived from individual cells. Scale bar: 4 μm. P = perimeter; A = area; C = circularity.

The perimeter deviation was used to compute the riffle-index (R-index), a continuous measure of junctional irregularity (Fig 5C). To ensure that the smoothing procedure did not introduce systematic distortion of basic morphological measurements, we quantified agreement between original and smoothed cell objects across 5,306 paired cells. No relevant differences were observed for cell area, length, or width, and agreement analysis demonstrated minimal bias and negligible error relative to biological variability (S5C–S5E Fig, S1 Text).

Although manual tracing-based approaches have been used to quantify junctional ruffling [11,23,24], these procedures are labor-intensive and observer-dependent, and no widely adopted quantitative gold standard exists. Therefore, we compared the automated R-index to blinded visual phenotyping at the image level. Immunofluorescence images of WT, *PKD2* ⁻ ^/^ ⁻ , and *PKD2^-/-^* rescue cells were assigned to genotype in a blinded manner based on junction appearance (riffled vs. intermediate vs. smooth). In total, 108 images were classified, and 80.6% were assigned correctly (87/108; confusion matrix in S1 Text). Misclassifications occurred predominantly between WT and *PKD2^-/-^* rescue, consistent with the rescue phenotype appearing highly similar to WT by visual inspection. We then computed the R-index from the same images and found that it reproduced the qualitative ordering observed in the blinded assessment: *PKD2* ⁻ ^/^ ⁻ showed the lowest R-index values, WT the highest, and *PKD2^-/-^* rescue intermediate, with values closer to WT (Fig 6A). In contrast, cell area did not differ across groups (Fig 6B). Together, these results indicate that the R-index provides an objective, scalable measure of junctional meandering that aligns with blinded visual phenotyping and enables high-throughput comparisons across large datasets and genotypes.

**Fig 6:**
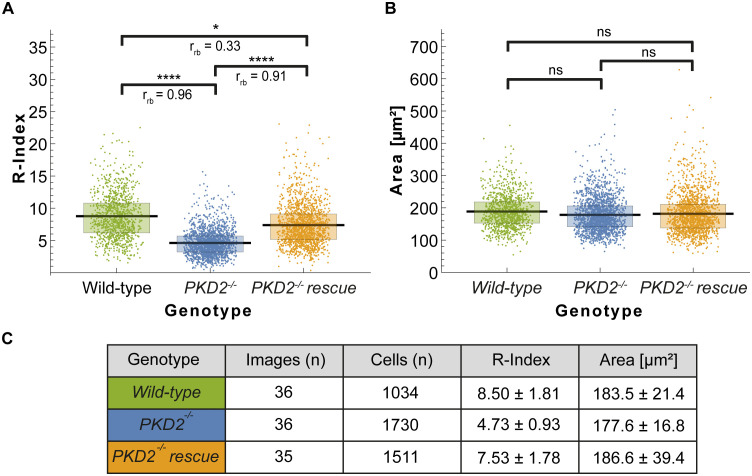
Data visualization in a high throughput setting. **(A)** Distribution of R-indices across genotypes. Each datapoint represents an individual cell. The black horizontal bar indicates the arithmetic mean, and boxes represent the 25th to 75th percentile. Statistical testing was performed on per-image-file aggregated values. Genotype had a significant effect (Kruskal-Wallis test, H(2) = 64.86, p < 0.001). Pairwise comparisons were significant between all groups after correction (p < 0.001). **(B)** Distribution of cell area across genotypes, visualized as in **(A)**. No significant differences were observed (Kruskal-Wallis test, H(2) = 1.98, p = 0.375; all pairwise comparisons ns). **(C)** Summary statistics including number of image files and number of cells. Aggregated R-Index and area are shown as mean ± SD. ns: not significant; *: p_adj_ ≤ 0.05; ****: p_adj_ ≤ 0.0001. Effect sizes are reported as rank-biserial correlation (r_rb_).

### Quantification of cytoskeletal traction between different phenotypes

To investigate whether riffled junction patterns are associated with altered mechanical coupling across cell-cell borders, we derived a border-level proxy for cytoskeletal traction from the segmented tight-junction network. After skeletonization, the junction graph was decomposed into border segments formed by neighboring cell pairs. For each border segment, we quantified its lateral deflection by computing the enclosed area between the segmented border polyline and the straight chord connecting the same endpoints (S6C Fig). The straight chord serves as a reference between endpoints while the enclosed area quantifies the deviation of the segmented border from this reference. Because this enclosed area corresponds to the integral of border displacement along the segment, it serves as a simple geometric surrogate for junctional deformation and, by extension, as a traction proxy for asymmetric force transmission along the shared border. This interpretation assumes that directional border deflection reflects an imbalance in mechanical coupling across the shared interface and does not represent a direct force measurement.

We applied this framework to co-culture experiments in which WT.GFP cells were mixed with *CGN ⁻* ^*/*^ *⁻* cells. CGN encodes cingulin, a tight-junction-associated scaffold protein involved in junction–cytoskeleton coupling. Similar to *PKD2 ⁻* ^*/*^ *⁻* cells, *CGN ⁻* ^*/*^ *⁻* cells exhibit reduced junctional meandering. Co-cultures generate heterotypic WT-mutant interfaces within the same epithelial field, enabling direct comparison of homotypic and heterotypic borders under identical imaging conditions and allowing directional analysis relative to WT using the GFP channel (Fig 7A and 7B). The R-index differed systematically by border pairing (*CGN-CGN* < *CGN*-WT < WT-WT; Fig 7C). Moreover, the enclosed-area traction proxy revealed a directional bias at heterotypic borders: WT-CGN borders showed a significant shift toward WT, whereas WT-WT borders showed no directional bias (Fig 7D). Together, these results provide a practical example of how the border-based traction proxy can detect genotype-dependent differences in force transmission across cell-cell contacts that are difficult to quantify reliably by visual inspection alone.

**Fig 7:**
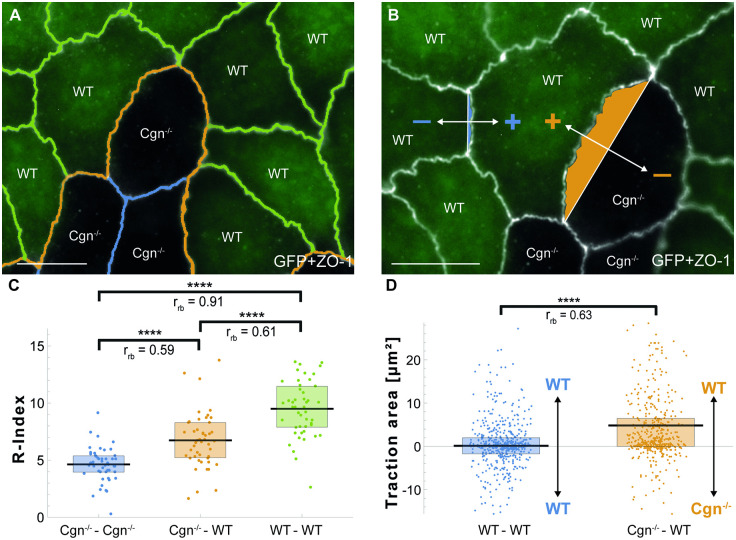
Estimating cytoskeletal traction at shared cell borders in co-culture experiments. **(A)** Co-culture cells were assigned to genotype using GFP signal (WT: GFP ⁺ ; *CGN ⁻* ^*/*^ *⁻* : GFP⁻). The segmented tight junction network was subdivided into border segments between adjacent cell pairs (color-coded by border identity). **(B)** Traction proxy calculation: cytoskeletal traction at shared borders was estimated from the signed enclosed area of each border segment, with positive values predefined as deflection toward WT cells and negative values as deflection toward the opposing cell. For WT-WT borders, orientation was assigned randomly. **(C)** R-index by border genotype pairing (*CGN*-*CGN*; *CGN*-WT; WT-WT). Each datapoint represents one image file. Black bar = mean; boxes = 25th-75th percentile. Mean ± SD: *CGN*-*CGN* 4.63 ± 1.50 (n = 48), *CGN*-WT 6.74 ± 2.42 (n = 47), WT-WT 9.50 ± 2.43 (n = 47). Genotype pairing had a significant effect (Kruskal-Wallis test, H(2) = 71.57, p < 0.0001). Pairwise comparisons (Mann-Whitney U with Holm correction) were significant (all p_adj_ < 0.0001; effect sizes r_rb_: *CGN*-WT vs *CGN*-*CGN* = 0.59, WT-WT vs *CGN*-*CGN* = 0.91, WT-WT vs *CGN*-WT = 0.61). **(D)** Enclosed area (traction proxy) per border segment, where each datapoint represents an individual segment: WT-WT (n = 560; 0.13 ± 7.86 µm²) showed no directional bias, whereas WT-*CGN* (n = 511; 4.82 ± 12.04 µm²) showed a directional bias toward the WT cell (Mann-Whitney U: p < 0.0001, r_rb_ = 0.63). **** p_adj_ < 0.0001. Effect sizes are rank-biserial correlation (r_rb_). WT = wild-type, *CGN* = Cingulin.

### Optimization for scalability and high throughput screening

The entire image processing pipeline was optimized for high-throughput screening. User interaction was minimal and limited to image selection, parameter specification, and a brief post-analysis quality control step to detect gross systematic segmentation errors and samples compromised by technical staining artifacts. We successfully processed datasets exceeding 3,000 images and more than 100,000 cell objects to screen a range of genotypes associated with the ADPKD spectrum. By comparison, a qualitative blinded manual classification of a typical dataset of around 100 images into broad phenotype categories required approximately 1 h, but did not provide object-based quantitative measurements. Establishing the DL-supported workflow required a one-time annotation effort of approximately 20–25 h for the training set, after which large datasets could be analyzed in a standardized batch-wise manner. In addition to handling large datasets, individual image tiles were automatically stitched together to generate larger composite images (S7 Fig), reducing field-of-view selection bias and increasing the number of analyzable cells per condition.

### Transfer of the workflow to other epithelial imaging datasets

To assess technical compatibility beyond MDCK monolayers, we applied the workflow to immunofluorescence images of Drosophila egg chamber epithelia stained for β-catenin, a marker of adherens junctions [35]. While these images could be processed by the pipeline, segmentation quality was not sufficient for robust fully automated analysis without dataset-specific optimization, and under-segmentation was observed in the representative example shown in S8 Fig. Reliable application to new cell types or imaging conditions would require additional fine-tuning using representative training images and systematic validation, which is expected to keep the required effort modest.

## Discussion

Cell shape is a fundamental determinant of tissue architecture and organ morphogenesis [36]. In epithelia, cell geometry emerges from the interplay between cytoskeletal tension and junctional organization [1,3]. Disruption of this balance contributes to disease, including cystic kidney disease. In ADPKD, cystogenesis is associated with a shift from cuboidal to flattened epithelial morphology, a process that can be reversed by re-expression of *PKD* genes [37,38]. Quantitative analysis of epithelial morphology is therefore not only descriptive, but can provide mechanistic insight into how disease-associated mutations alter epithelial organization.

Quantitative analysis of epithelial morphology already builds on a substantial body of image-analysis methods. Established image-analysis platforms such as CellProfiler, Cellpose or Tissue Analyzer support segmentation, feature extraction, and epithelial morphometry [28,29,33,39], while dedicated tools such as TISMorph, Junction Mapper, and Polarity-JaM provide complementary descriptors of boundary irregularity, cell-cell contact organization, marker intensity, interface linearity and polarity [30–32]. These tools overlap conceptually with our analysis. However, our aim was not to replace existing platforms, but to derive assay-specific readouts for fine junctional meandering in ZO-1-stained monolayers and genotype-resolved behavior at shared borders in mixed-cell experiments, a combination not directly supported by the evaluated tools.

Within this framework, the R-index provides more than an automated restatement of visual impression. In our ADPKD model, it captured genotype-associated differences in junctional morphology in a manner consistent with visual assessment, whereas conventional descriptors such as cell area were less informative. This is important because it indicates that junctional meandering represents a phenotype that is not reducible to coarse changes in cell size or simple shape descriptors. The biomedical value of the R-index lies in its ability to quantify genotype-associated alterations in junctional architecture as a continuous and scalable phenotype. Because it is derived from a smoothed contour model, its absolute value depends on the chosen smoothing procedure; in the present study, identical settings were applied across all conditions so that relative differences could be compared consistently. The relevance of specific R-index differences will therefore depend on the experimental context. In practical terms, this makes it useful for comparing mutants, rescue conditions, or perturbation experiments across large datasets, and suggests that it may serve as a sensitive endpoint for image-based phenotypic profiling in ADPKD-related epithelial models [40,41].

The traction proxy extends this analysis from phenotype description toward a first approximation of epithelial mechanics. By decomposing the segmented junction network into individual shared borders and quantifying directional border deflection, the model provides a geometry-based surrogate for asymmetric force transmission at cell-cell interfaces. We view this readout as a complementary alternative for situations in which direct biophysical force measurements are too labor-intensive or insufficiently scalable for rapid analysis of large co-culture datasets [42]. Nevertheless, in co-culture settings, it offers an additional layer of information that is difficult to extract reproducibly by visual inspection alone. A key advantage of this approach lies in its ability to reveal genotype-dependent differences in intercellular mechanical coupling at the level of individual borders within the same epithelial field. This may be particularly informative in mosaic systems, rescue experiments, or co-culture assays, where phenotypes emerge specifically at heterotypic interfaces rather than uniformly across the monolayer.

A further strength of the workflow is its scalability. Manual quantification of junctional morphology has been informative in previous studies, but is difficult to scale and standardize across large datasets [7,10,11,43,44]. In this context, the present workflow enables assay-specific phenotyping of junctional meandering and shared-border behavior at substantially larger scale and reduces user-induced selection and confirmation bias. This is especially relevant in ADPKD research, where image-based phenotyping is increasingly being integrated into larger perturbation and screening frameworks [41]. At the same time, systematic errors remain an inherent risk of automated workflows [45]. We therefore applied conservative quality-control criteria to reduce the inclusion of incorrectly segmented cells. Because cell numbers in this system can be increased relatively easily through additional image fields or biological replicates, we considered this trade-off appropriate in favor of detection quality. For this reason, manual review remains advisable when the pipeline is transferred to substantially different imaging conditions or biological systems.

The workflow may in principle be adaptable to other junctional markers or epithelial imaging settings, provided that the marker of interest yields sufficiently continuous junctional contours. Similar junctional phenotypes have also been described with other markers, including ZO-2/ZO-3, actin, cingulin, and E-cadherin, suggesting that the underlying analytical framework may be transferable to related readouts [6,7,10,11,23,46]. Potential target systems include other epithelial monolayers, organoid-derived intestinal epithelia, and developmental epithelia with well-resolved junctional labeling [47,48]. However, successful transfer will depend on image quality, marker continuity, staining characteristics, and dataset-specific retraining or validation. While transfer to related settings should in principle be feasible through retraining of the U-Net on a limited set of representative images, this will need to be established for each marker and experimental context.

Several limitations should nevertheless be acknowledged. First, the workflow operates on 2D projections and therefore does not capture three-dimensional epithelial organization or extracellular matrix interactions known to influence cystogenesis in ADPKD [49]. Extending the approach to organoids or other 3D systems would likely improve pathophysiological relevance, but would also require adaptations in segmentation and morphometric modeling. Second, our traction estimates are indirect and should ideally be validated against direct force measurements such as traction force microscopy [50]. Third, although the full pipeline can be implemented with widely available tools, its more specialized modules still require customization beyond general-purpose image analysis systems. State-of-the-art software provides a strong basis for segmentation and basic morphology analysis, whereas assay-specific analysis of junctional meandering and shared-border mechanics is likely to continue to benefit from custom implementations.

In summary, we present a deep learning-supported workflow for quantitative analysis of epithelial morphology that combines robust segmentation with readouts tailored to junctional meandering and shared-border mechanics. In our ADPKD model, the R-index provides a scalable phenotype of genotype-associated junctional remodeling, while the border-based traction proxy highlights differences in intercellular force transmission that are difficult to assess reliably by manual inspection alone. Our findings highlight the power of automated image analysis to move beyond manual inspection, providing deeper, data-driven insight into the complex interplay between cell shape, mechanics, and function in epithelial biology, with implications for disease pathogenesis and morphogenetic regulation.

## Materials and Methods

### Cell lines

Madin-Darby Canine Kidney cells (MDCK) (American Type Culture Collection, Manassas, USA) as well as genetically modified *PKD1*^*-/-*^*, PKD2*^*-/-*^, *PKD2*^*-/-*^ rescue, *LIMA1*^*-/-*^*, MYO1C*^*-/-*^*, CGN*^*-/-*^, and WT.GFP lines, were cultivated as adherent monolayers in Dulbecco’s modified Eagle’s medium (Thermo Fisher Scientific Inc., Waltham, USA) supplemented with 10% heat-inactivated fetal bovine serum (Sigma-Aldrich, St. Louis, USA). Cell lines were maintained in a humidified incubator at 37°C with 10% CO_2_ and passaged every 2–3 days using 0.25% trypsin-EDTA (Thermo Fisher Scientific Inc., Waltham, USA).

### Immunofluorescence staining

Immunofluorescence staining of MDCK cells was performed as previously described [51]. Briefly, cells were seeded on cover glasses (Carl Roth) in six-well plates (Greiner Bio-One, Germany) and grown for ten days. The α-ZO-1 primary antibodies (Santa Cruz Biotechnology, Dallas, USA and Thermo Fisher Scientific Inc., Waltham, USA) were diluted 1:50 or 1:500 in PBS, respectively. Imaging was performed on an Axio Observer.Z1 microscope (Zeiss, Jena, Germany) equipped with a Zeiss i-Plan-Apochromat 63x/1.40 Oil DIC M27 objective using ZEN 2 (blue edition) Version 2.0.0.0. Pixel size: 0.102 µm/px. Tile functions were used to generate stitched images.

### Co-culture experiments

For co-culture assays, WT.GFP cells were mixed 1:1 with cells of the indicated genotype prior to seeding. WT.GFP cells were identified via the GFP channel when overlap was > 50% (GFP-positive), while the second genotype was identified as GFP-negative. All subsequent cultivation, immunofluorescence staining and imaging were performed identically to the monoculture experiments described above.

### *Drosophila* stocks and genetics

*Drosophila melanogaster* stock *w[118]* was used. Stock was maintained on standard fly food (10 L water, 74.5 g agar, 243 g dry yeast, 580 g corn meal, 552 mL molasses, 20.7 g Nipagin, 35 mL propionic acid) at 22 °C. Flies were fed yeast paste for 48–72 h prior to dissection.

### Immunohistochemistry and imaging of *Drosophila* egg chambers

Ovaries were dissected and fixed in 4% paraformaldehyde/PBS for 15 min at 22°C. Washes were performed in PBS  +  0.1% Triton X-100 (PBT). Samples were incubated with primary antibody mouse anti-β-catenin (1:100, DSHB, N27A1) in PBT overnight at 4°C. Ovaries were washed with PBT and then incubated with dyes and secondary antibodies for 2 h at 22 °C. DAPI (1:500, Sigma), Phalloidin (1:500 Alexa Fluor 488) were used to visualize DNA and filamentous Actin, respectively. Donkey anti-mouse Alexa Fluor647 (Abcam, AB150111, 1:500) was used as secondary antibody. Samples were mounted using Molecular Probes Antifade Reagents and imaged using the Leica TCS SP8 confocal microscope.

### Training the deep learning network

The U-Net model was trained using the ImageJ U-Net plugin [26]. Image annotation was conducted in 3D Slicer [52] following the general instructions provided by Falk et. al. [26]. To ensure consistent ground-truth generation, a predefined segmentation strategy was established before annotation. For labeling, images were normalized for display (contrast/brightness adjustment for visual consistency) to achieve a comparable visual appearance across experiments. Tight junctions were annotated with a target width of approximately 3–4 pixels in well-focused regions.

Out-of-focus image regions were identified manually during annotation based on predefined morphological criteria, including (i) apparent widening of the tight junction signal by >100% compared with adjacent in-focus regions and/or (ii) clear straightening/loss of the characteristic meandering cell border appearance. Such regions, as well as background noise and non-junctional signal, were annotated as negative class and were therefore explicitly included in training. This strategy was used to reduce interference of blurred structures with downstream segmentation and morphometric analysis.

Annotation was performed by one trained doctoral student with experience in immunofluorescence image analysis. All labels underwent a second reading by a supervisor with >10 years of experience in immunofluorescence microscopy/image analysis, and discrepancies were resolved by consensus using the predefined segmentation criteria. Final label masks were converted to ImageJ (U. S. National Institutes of Health, Bethesda, Maryland) regions-of-interest to match the plugin requirements.

Training images were randomly selected from biologically independent experiments to minimize selection bias and improve generalizability. The dataset was assembled to capture a broad range of image appearances, including variation in signal intensity, background noise, staining quality, and tight junction meandering strength. Images from five genotypes (WT, *PKD2*^*-/-*^, *LIMA1*^*-/-*,^ *MYO1C*^*-/-*^, and *CGN*^*-/-*^), multiple exposure settings, different secondary antibodies, and four independent researchers were included. Full field-of-view images were used, but annotation was sparse (i.e., only selected regions/cell objects were labeled).

The final dataset comprised 9 training images (containing approximately 80 fully labeled cell objects), 3 validation images, and an independent test set of 3 images. No cross-validation was performed.

Training was conducted using the “Training from scratch” option with the “2D Cell Net (v0)” model [26] and the following settings: element size, 0.102 µm × 0.102 µm; input patch size, 1020 × 1020 px; learning rate, 1 × 10 ⁻ ⁴; and 10,000 epochs. Computation was performed on a remote server running Ubuntu 16.04.6 LTS (Canonical, London, UK) using the precompiled U-Net library with a Nvidia (Santa Clara, California) Geforce GTX 1080 Ti (12GB VRAM).

For model evaluation, segmentation performance was assessed on the independent test set using overlap-based metrics (intersection over union (IoU) and Dice coefficient) as well as pixel-wise classification metrics (precision (positive predictive value) and recall (sensitivity)), all computed on annotated pixels. In addition, segmentation outputs were visually reviewed after quantitative analysis to identify potential systematic errors and to verify sufficient exclusion of out-of-focus regions prior to downstream morphometric analysis.

### Image analysis

A custom Fiji ImageJ (version 1.53b) script was developed for automatic pre-processing and segmentation, enabling high throughput analysis. Subsequent analysis steps were implemented in Mathematica 12.0 (Wolfram Research, Champaign, Illinois). The source code, sample data and documentation are available on GitHub at https://github.com/JahnJ/U-Net-based-image-quantification-of-epithelial-cell-shapes/. We have also used Zenodo to assign a DOI to the repository: 10.5281/zenodo.15857671.

### Statistical analysis

Statistical analyses were performed in Wolfram Mathematica (12.0). Individual cells were treated as observational units for visualization, and measurements were aggregated per image file for statistical testing to ensure independence of experimental units and avoid pseudoreplication. Normality was assessed using the Shapiro-Wilk test and visual inspection of distributions; as data were not normally distributed, non-parametric tests were used. Differences across genotypes were assessed using the Kruskal-Wallis test, followed by pairwise Mann-Whitney U tests. P-values were adjusted for multiple comparisons using the Holm method. Effect sizes were reported as rank-biserial correlation (r_rb_). Statistical significance was defined as (adjusted) p < 0.05. Descriptive data are presented as mean ± SD.

## Supporting information

S1 Text Supplementary Methods and Results for additional analysis. (DOCX)

S1 Fig Meandering in ADPKD-related genotypes. Immunofluorescence images of MDCK WT (A), *PKD1*^*-/-*^ (B), *PKD2*^*-/-*^ (C) and *PKD2*^*-/-*^*rescue* (D) cells, which were stained with an α-Zonula occludens 1 primary antibody and a Cy3-conjugated secondary antibody. (A) WT cells show prominent membrane riffles. (B/C) *PKD1*^*-/-*^ and *PKD2*^*-/-*^ cells exhibit smooth cell borders with minimal indentations. (D) Re-expression of *PKD2* restores the meandering morphology of tight junctions. Scale bar: 10 µm. MDCK = Madin-Darby Canine Kidney; *PKD1* = Polycystic kidney disease gene 1; *PKD2* = Polycystic kidney disease gene 2; WT = Wildtype; ZO-1 = Zonula occludens protein 1.
(TIF)

S2 Fig Subtle visual differences in tight junction meandering between genotypes. Exemplary immunofluorescence images of MDCK WT (A), *PKD2*^-/-^ (B) and *PKD2*^-/-^rescue (C) cells stained for Zonula occludens protein 1 using a Cy3-conjugated secondary antibody. Tight junctions in all three genotypes exhibit a comparable polygonal organization with varying degrees of membrane undulation. Although *PKD2* loss and re-expression are associated with alterations in junctional morphology, these differences could be visually subtle and not readily distinguishable by qualitative inspection alone. In a blinded assessment, discrimination between genotypes based solely on apparent tight junction meandering would therefore be challenging, underscoring the need for quantitative analysis. Scale bar: 10 µm. MDCK = Madin-Darby Canine Kidney; *PKD2* = Polycystic kidney disease gene 2; WT = Wildtype; ZO-1 = Zonula occludens protein 1.
(TIF)

S3 Fig Training procedure for the ZO-1 deep learning model. (A) Representative immunofluorescence image with manually annotated tight junction structures used for training. (B) Annotated images were split into a training and validation dataset and used to train a U-Net convolutional neural network. The displayed parameters (learning rate and number of iterations) can be adjusted as needed. Once trained, the ZO-1 segmentation model can be applied to new image data for automated analysis. Panel (B) is adapted from [26]. Scale bar: 10 µm. ZO-1 = Zonula occludens protein 1.
(TIF)

S4 Fig Detection of individual cell objects from tight junction staining. (A) MDCK cells were fixed, stained with ZO-1 antibodies, and imaged using immunofluorescence microscopy to visualize tight junctions. (B) The trained U-Net deep learning model was applied to segment tight junctions. Regions with insufficient signal or out-of-focus areas were excluded to maintain segmentation accuracy. (C) Individual cell objects were extracted from the segmentation using an adapted watershed algorithm. Area values are shown for each detected object. The full sample contains 45 detected cells; a cropped region is shown for improved visibility. (D) Multiple morphological parameters, such as area and perimeter, can be calculated for each cell object. Scale bar: 10 µm (A-C). MDCK = Madin-Darby Canine Kidney; ZO-1 = Zonula occludens protein 1.
(TIF)

S5 Fig Effect of smoothing on cell morphology and quantitative agreement with original measurements. (A) Representative example of original riffled cell objects. (B) Corresponding smoothed cell objects after application of the smoothing procedure. (C-E) Bland-Altman analysis comparing measurements obtained from original and smoothed objects for (C) cell area, (D) cell length, and (E) cell width. Differences between paired measurements are plotted against their mean. Solid lines indicate the mean difference (bias), and dashed lines indicate the 95% limits of agreement (mean difference ± 1.96 SD). Bias and limits of agreement were small for all parameters (area: bias = 0.143 µm^2^, limits −1.085 to 1.371 µm^2^; length: bias = −0.048 µm, limits −0.228 to 0.132 µm; width: bias = −0.016 µm, limits −0.149 to 0.117 µm), indicating that smoothing preserves morphological measurements with negligible quantitative error. For visualization, the y-axis range in panels C-E was restricted to emphasize the agreement region (bias and 95% limits of agreement).
(TIF)

S6 Fig Genotype-specific cell interaction analysis in mixed cultures. (A) GFP channel of a mixed culture of GFP-labeled WT and unlabeled *CGN*^*-/-*^ MDCK cells. GFP-positive cells correspond to WT cells. (B) Overlay of the GFP signal with cell objects segmented from the corresponding ZO-1 image. Cells were classified as GFP-positive (WT, green) when more than 50% of the segmented cell area overlapped with the binarized GFP signal. Otherwise, they were classified as GFP-negative (*CGN*^*-/-*^, gray). (C) Cropped region indicated in (B), showing the skeletonized ZO-1 junction network used for border extraction. (D) Separation of the skeletonized junction network into individual border segments. The network was split at branch points, and each border segment was assigned to the two adjacent cells and classified according to genotype pairing. (E) Example of two neighboring cells after genotype assignment. The WT cell is shown in green, the *CGN*^*-/-*^ cell in gray. The enclosed area between their junctions was calculated from segment endpoints and used as a proxy for traction. In the example shown, traction is directed toward the WT cell. Scale bar: 10 µm. *CGN* = Cingulin; GFP = Green fluorescent protein; WT = Wildtype; ZO-1 = zonula occludens 1.
(TIF)

S7 Fig Scalability for dataset expansion through image stitching. (A) Representative subregion of the large immunofluorescence image shown in (B) depicting MDCK WT cells stained for ZO-1. (B) Full automatically stitched image generated from a 9 × 9 tile scan acquired with a 40 × objective using an Alexa Fluor 488-labeled secondary antibody. Image stitching was executed directly at the microscope, resulting in a substantial increase in imaged area and data volume. Cell object detection was performed following stitching. Total processing time was approximately 3 h. Large black regions indicate insufficient staining or out-of-focus areas and were excluded from analysis. The white box denotes the region shown in (A). Scale bars: 25 µm (A), 150 µm (B). MDCK = Madin-Darby Canine Kidney; ZO-1 = Zonula occludens protein 1; WT = Wildtype.
(TIF)

S8 Fig Transfer of the image analysis pipeline to *Drosophila* egg chamber epithelia. (A) Maximum-intensity projection of a *Drosophila* egg chamber immunofluorescence image stained for β-catenin to label adherens junctions of the follicle epithelium. (B) Segmentation output obtained after applying the pipeline to the same image. The arrow indicates an example of under-segmentation, where adjacent cells were erroneously merged, illustrating that dataset-specific fine-tuning is required for robust analysis**.** Scale bars: 25 μm.
(TIF)

S9 Fig Representative segmentation comparison across state-of-the-art tools. Representative ZO-1-stained epithelial images with segmentation overlays from the U-Net pipeline (this study), Cellpose-SAM, Tissue Analyzer, and CellProfiler threshold-based segmentation.
(TIF)
