# Supplementary material for: Deep learning-supported image quantification of epithelial cell shapes and its application to polycystic kidney disease
Source: PLoS Comput Biol. 2026 Aug 3;22(8):e1014614. doi: 10.1371/journal.pcbi.1014614 (PMC13456487; doi:10.1371/journal.pcbi.1014614)
Supplement: Supplementary file 1 [file pcbi.1014614.s001.docx]

Supplementary Text S1

Deep learning-supported image quantification of epithelial cell shapes and its application to polycystic kidney disease

Johannes Jahn, Alexis Hofherr, Clara Consoli, Berenike Fajen, Rebekka Goll, Greta Theresa Liedtke, Adrian Boehm, Friederike Selbach, Paul Christoph Zeisler, Vanessa Weichselberger, Anne-Kathrin Classen, Lukas Westermann, Tilman Busch, Michael Köttgen

Content

[Supplementary methods	2](#_Toc230298644)

[Benchmarking of segmentation and analysis workflows	2](#_Toc230298645)

[Statistical analysis: Smoothed modeling	3](#_Toc230298646)

[Supplementary results	3](#_Toc230298647)

[Segmentation and analysis results of state-of-the-art tools	3](#_Toc230298648)

[Runtime comparison	3](#_Toc230298649)

[Qualitative segmentation behavior relevant to meandering quantification	4](#_Toc230298650)

[Qualitative segmentation assessment	4](#_Toc230298651)

[Qualitative comparison with dedicated morphometric and junction-analysis tools	5](#_Toc230298652)

[Summary of suitability for the screening workflow	6](#_Toc230298653)

[Agreement between riffled and smoothed cell measurements	7](#_Toc230298654)

[Confusion matrix of blinded visual genotype classification based on tight-junction morphology	7](#_Toc230298655)

[References	8](#_Toc230298656)

# Supplementary methods

## Benchmarking of segmentation and analysis workflows

To assess whether established segmentation and morphology-analysis tools are suitable for our ZO-1 tight-junction images and, critically, for tight-junction meandering quantification, we benchmarked three widely used tools against our specific task: Tissue Analyzer v1.06a (Python implementation) [1], executed on a NVIDIA (Santa Clara, California) GeForce RTX 3070 (8 GB VRAM); Cellpose v4.0.8 (Cellpose-SAM, Python implementation) [2], executed on NVIDIA (Santa Clara, California) GeForce RTX 3070 (8 GB VRAM); CellProfiler v4.2.8 (Standalone) [3]. Benchmarking was performed on N = 108 ZO-1-stained epithelial images (wild-type, *PKD2^-/-^*, *PKD2^-/-^* rescue). All images were converted to TIF and normalized prior analysis. The comparison was designed to evaluate (i) segmentation behavior relevant to meandering analysis, (ii) robustness across heterogeneous imaging conditions, and (iii) handling of out-of-focus areas (defined as apparent widening of the tight junction signal by >100% compared with adjacent in-focus regions and/or clear straightening/loss of the characteristic meandering cell border appearance) not suitable for adequate meandering quantification. In addition to qualitative assessment, we evaluated runtime, defined as wall-clock time, and quantitatively assessed false inclusion of unsuitable cell objects. False inclusion was defined as the detection of a segmented cell object in a region meeting our predefined out-of-focus criteria or showing other relevant segmentation errors, such as cell fusion, rendering the object unsuitable for reliable meandering quantification.

In addition to the segmentation-focused benchmarking, we performed a qualitative comparison of the dedicated morphometric and junction-analysis tools TISMorph [4], Junction Mapper [5], and Polarity-JaM [6] to assess segmentation strategy, descriptors for junctional morphology and support for genotype-resolved shared-border analysis in mixed-cell experiments.

## Statistical analysis: Smoothed modeling

Agreement between morphological measurements obtained from original riffled and smoothed cell objects was assessed using complementary statistical approaches. Pearson correlation coefficients were calculated to quantify the linear association between paired measurements. To evaluate absolute agreement and detect potential systematic bias, Bland–Altman analysis was performed by plotting the difference between paired measurements against their mean. The mean difference (bias) and 95% limits of agreement (mean difference ± 1.96 × standard deviation of the differences) were computed. To quantify the magnitude of deviations, the root mean square error (RMSE) was calculated, and normalized RMSE (NRMSE) was obtained by dividing RMSE by the measurement range. Bias and error metrics were interpreted relative to the intrinsic variability of the data, quantified by the standard deviation and median of the original measurements. No hypothesis testing was used to assess differences, as large sample sizes can yield statistically significant results even for negligible deviations. Instead, agreement was evaluated using effect size–based metrics and agreement analysis, which provide a more appropriate assessment of measurement fidelity. All analyses were performed using Wolfram Mathematica 12.0 (Wolfram Research, Champaign, Illinois). Measurements were treated as paired observations, and all available data points were included.

# Supplementary results

## Segmentation and analysis results of state-of-the-art tools

### Runtime comparison

Total processing times for N = 108 images were:

- U-Net segmentation (this study): 5:45 min (345 s; 3.19 s/image)
- Cellpose-SAM: 12:36 min (756 s; 7.00 s/image)
- Tissue Analyzer: 45:00 min (2700 s; 25.0 s/image)
- CellProfiler (threshold-based): 1:20 min (80 s; 0.74 s/image)
- Downstream Mathematica analysis: 2:36 min (156 s; 1.44 s/image)

These results show that CellProfiler thresholding is fastest for generating tight junction segmentation, while deep learning-based segmentations tend to take longer, depending on the available GPU power.

### Qualitative segmentation behavior relevant to meandering quantification

Across representative ZO-1 images, we observed consistent tool-dependent trade-offs:

Cellpose-SAM produced strong overall detection of junctional structures without further user input, but frequently smoothed fine boundary undulations, reducing preservation of the meandering configuration. In addition, out-of-focus regions were mostly consistently segmented, which can bias downstream meandering metrics unless unreliable regions are manually excluded.

Tissue Analyzer yielded excellent segmentation quality out of the box and was robust to background noise and intensity variation. Fine meandering patters were sufficiently segmented. However, as with Cellpose-SAM, out-of-focus regions were partly segmented as valid, requiring manual exclusion to prevent unreliable meandering measurements in the downstream pipeline.

CellProfiler provided flexible classical segmentation and feature extraction, but segmentation quality was less consistent across heterogeneous images, reflecting sensitivity to fixed thresholds and processing parameters. This sensitivity was most apparent under varying signal intensity where the tight junction was segmented as a bold border, missing fine meandering patterns.

A side-by-side visual comparison is provided in S9 Fig.

### Qualitative segmentation assessment

To further assess the suitability of the tested segmentation approaches for downstream tight-junction meandering analysis, we applied our analysis workflow to the segmentation outputs of Tissue Analyzer and CellProfiler. Cellpose-SAM was included in the qualitative benchmarking but was not carried forward to downstream quantification because its segmentation output was not considered reliable for tight-junction meandering analysis, primarily due to smoothing of fine junctional undulations and inclusion of out-of-focus regions (S9 Fig).

Downstream analysis of Tissue Analyzer and CellProfiler outputs was possible without major adaptation. To specifically evaluate the handling of out-of-focus regions, we manually reviewed a representative subset of n = 12 images and quantified how many detected cell objects were falsely included despite fulfilling our predefined out-of-focus criteria, and would therefore bias meandering quantification.

Across this subset, Tissue Analyzer detected the highest number of cell objects (674 total; 56.2 ± 5.7 per image), followed by CellProfiler (459 total; 38.3 ± 8.6 per image) and the U-Net pipeline (439 total; 36.6 ± 10.8 per image). However, Tissue Analyzer also showed the highest number of falsely included cells (229 total; 19.1 ± 7.7 per image; 34.0% of detected cells), whereas CellProfiler produced fewer false inclusions (63 total; 5.3 ± 2.9 per image; 13.7%) and the U-Net pipeline the lowest number (22 total; 1.8 ± 1.4 per image; 5.0%).

After excluding falsely included cells, the number of potentially valid analyzed cells was 445 total (37.1 ± 9.3 per image) for Tissue Analyzer, 396 total (33.0 ± 9.0 per image) for CellProfiler, and 417 total (34.8 ± 10.4 per image) for the U-Net pipeline. Thus, although Tissue Analyzer yielded the highest raw detection count, a substantial fraction of these detections originated from out-of-focus or otherwise unsuitable regions. In contrast, the U-Net pipeline was more conservative with respect to out-of-focus regions while still preserving a similar number of valid cell objects for downstream analysis. These results support the use of the task-specific U-Net segmentation for reliable tight-junction meandering quantification under heterogeneous imaging conditions.

### Qualitative comparison with dedicated morphometric and junction-analysis tools

To further position our workflow, we qualitatively compared it with existing tools for cell-shape and junction morphometry. This comparison focused on specific junctional parameters, segmentation strategies, and support for shared-border analysis in mixed-cell experiments.

TISMorph provides a broad set of single-cell morphology, texture, spreading, and boundary-irregularity descriptors, including measures related to boundary waviness and roughness. These features are conceptually related to our aim of quantifying contour irregularity. However, TISMorph relies on supervised threshold-based segmentation and CellProfiler-based cell detection, followed by analysis of individual cell objects. It is therefore well suited for detailed single-cell morphometry, but does not directly support automated extraction of shared epithelial borders or genotype-resolved analysis of heterotypic interfaces in mixed cultures.

Junction Mapper is specifically designed for cell-cell contact analysis and provides junction-level descriptors including interface length, marker intensity, fragmentation, and interface linearity. The shown interface linearity index is conceptually related to contour-based measures such as our R-index. Junction Mapper is particularly useful for intensity- and fragmentation-based junction phenotypes, including cases with discontinuous staining. However, it does not include task-specific deep learning segmentation for our ZO-1 image data and does not directly provide genotype-resolved directional shared-border analysis in mixed-cell experiments.

Polarity-JaM offers a broad feature set for polarity, morphology, marker intensity, flow, and junction analysis. Its segmentation is based on Cellpose, and its junction features build on Junction Mapper. While this makes Polarity-JaM a powerful framework, the Cellpose-based segmentation has the same limitations discussed above for our ZO-1 dataset, particularly inclusion of out-of-focus regions and potential smoothing of fine junctional undulations. In addition, genotype-resolved mixed-culture border analysis is not available out of the box.

### Summary of suitability for the screening workflow

While all tested tools are valuable for general epithelial segmentation and specific morphology extraction, none natively supported the combination of (i) out-of-focus-aware segmentation aligned to our exclusion strategy, (ii) computation of a tight-junction meandering metric, and (iii) genotype-resolved shared-border analysis in co-culture. These constraints motivated the present task-specific pipeline.

## Agreement between riffled and smoothed cell measurements

Agreement between original riffled and smoothed cell measurements was assessed for the morphological parameters area, length, and width. The analysis was performed on a dataset comprising 146 images from five genotypes (Wildtype, *PKD2^-/-^*, *PKD2^-/-^* rescue, *LIMA1^-/-^*, *MYO1C^-/-^*), generated by three independent laboratory members. In total, 5,306 paired cell objects (original and corresponding smoothed objects) were included in the analysis. For cell area, the correlation was near-perfect (Pearson r = 0.99995), with a minimal bias of 0.143 µm² and narrow limits of agreement (−1.085 to 1.371 µm²) (S5C Fig). NRMSE was 0.11%, corresponding to only 0.08% of the median area (179.33 µm²) and negligible relative to the intrinsic variability (SD = 59.44 µm²). Similarly, cell length showed extremely high agreement (r = 0.999741), with a negligible bias of −0.048 µm and tight limits of agreement (−0.228 to 0.132 µm) (S5D Fig). NRMSE was 0.22%, representing only 0.25% of the median length (19.04 µm) and far below the natural variability (SD = 4.03 µm). Cell width measurements were likewise highly consistent (r = 0.999606), with minimal bias (−0.016 µm) and narrow limits of agreement (−0.149 to 0.117 µm) (S5E Fig). NRMSE was 0.35%, corresponding to 0.13% of the median width (12.71 µm) and negligible relative to the intrinsic standard deviation (SD = 2.42 µm). Together, these results demonstrate that the smoothing procedure preserves cell morphology measurements with near-perfect fidelity and introduces negligible systematic or random error relative to biological variability.

## Confusion matrix of blinded visual genotype classification based on tight-junction morphology

| True genotype | Classified as WT | Classified as PKD2 rescue | Classified as PKD2⁻/⁻ | Total |
| --- | --- | --- | --- | --- |
| WT | 28 | 8 | 0 | 36 |
| PKD2 rescue | 6 | 26 | 4 | 36 |
| PKD2⁻/⁻ | 1 | 2 | 33 | 36 |
| Total | 35 | 36 | 37 | 108 |

Rows indicate the true genotype after decoding; columns indicate the genotype assigned during blinded visual classification. Correctly classified: **87/108 = 80.6%.** Misclassifications between WT and PKD2 rescue accounted for 14/21 incorrect assignments.

# References

1. Aigouy B, Umetsu D, Eaton S. Segmentation and Quantitative Analysis of Epithelial Tissues. Methods Mol Biol. 2016; 1478:227–39. doi: 10.1007/978-1-4939-6371-3_13 PMID: 27730585.

2. Pachitariu M, Rariden M, Stringer C. Cellpose-SAM: superhuman generalization for cellular segmentation. bioRxiv. 2025. doi: 10.1101/2025.04.28.651001.

3. Stirling DR, Swain-Bowden MJ, Lucas AM, Carpenter AE, Cimini BA, Goodman A. CellProfiler 4: improvements in speed, utility and usability. BMC Bioinformatics. 2021; 22:433. Epub 2021/09/10. doi: 10.1186/s12859-021-04344-9 PMID: 34507520.

4. Alizadeh E, Xu W, Castle J, Foss J, Prasad A. TISMorph: A tool to quantify texture, irregularity and spreading of single cells. PLoS One. 2019; 14:e0217346. doi:10.1371/journal.pone.0217346.

5. Brezovjakova H, Tomlinson C, Mohd Naim N, Swiatlowska P, Erasmus JC, Huveneers S, et al. Junction Mapper is a novel computer vision tool to decipher cell-cell contact phenotypes. eLife. 2019;8:e45413. doi:10.7554/eLife.45413.

6. Giese W, Albrecht JP, Oppenheim O, Akmeriç EB, Kraxner J, Schmidt D, et al. Polarity-JaM: an image analysis toolbox for cell polarity, junction and morphology quantification. Nat Commun. 2025;16:1474. doi:10.1038/s41467-025-56643-x.
