# Supplementary material for: Deep learning-supported image quantification of epithelial cell shapes and its application to polycystic kidney disease
Source: PLoS Comput Biol. 2026 Aug 3;22(8):e1014614. doi: 10.1371/journal.pcbi.1014614 (PMC13456487; doi:10.1371/journal.pcbi.1014614)
Supplement: Supplementary file 2 [file pcbi.1014614.s018.docx]

**Response to the Editors’ and Reviewers’ Comments**

**Manuscript ID:** PCOMPBIOL-D-25-01691R1
**Title:** Deep learning-supported image quantification of epithelial cell shapes and its application to polycystic kidney disease

Dear Editors and Reviewers,

We thank the Editors and Reviewers for their careful evaluation of our manuscript and for their constructive and helpful comments. We have revised the manuscript accordingly, particular by expanding the discussion of related morphometric and junction-analysis tools. Below, we provide a point-by-point response with our changes to the text in blue.

**Reviewer #1:**

**Comment R1.1.1:** Reviewer reply: On the role of segmentation in quantification (line 104): The current phrasing implies that segmentation alone is necessary for quantification. It is worth noting that quantification does not always require segmentation (it is for shape quantification but not for image quantification); for instance, intensity-based or texture-based measurements can be performed directly on the raw image without prior segmentation.

***Response:*** *We thank the reviewer for this clarification. The respective passage has been revised as part of the changes described in response to R1.1.2, and the wording now refers specifically to our shape- and junction-morphology analysis rather than image quantification in general.*

*Introduction, page 5-6; line 107-113: “In addition, dedicated tools such as TISMorph, Junction Mapper and Polarity-JaM further provide morphometric and junction-related descriptors [30-32]. However, no single out-of-the-box workflow combines robust segmentation with exclusion of out-of-focus regions, quantification of tight-junction meandering, and in particular genotype-resolved analysis of border deflection as a proxy for traction forces at shared cell-cell interfaces. This highlights the need for a scalable workflow tailored to quantifying these junctional phenotypes.”*

**Comment R1.1.2:** On the claim of a lacking scalable workflow (line ~104): The statement "What remains lacking is a scalable workflow that preserves fine tight-junction contour irregularities and extracts descriptors tailored to this readout, including junction meandering and analysis of traction forces at cell-cell contacts" is not sufficiently supported and is directly challenged by existing tools such as Polarity-JaM (Nature Communications, 2025), which address different biological goals through similar approaches. The authors may legitimately argue that their method is simpler, faster, or more accurate for their specific use case (which I would absolutely agree with them) but claiming that such workflows are entirely lacking in the literature is an overstatement that a more comprehensive review of the current state of the art would correct.

***Response:*** *We thank the reviewer for this important comment. We agree that the previous wording may have overstated the lack of existing workflows. We therefore revised the Introduction to acknowledge dedicated morphometric and junction-analysis tools, including TISMorph, Junction Mapper, and Polarity-JaM, and reframed our contribution more specifically. Rather than claiming that such workflows are generally lacking, we now state that no single out-of-the-box workflow combines the requirements of our specific ZO-1-based ADPKD setting, namely robust exclusion of out-of-focus regions, tight-junction meandering quantification, and genotype-resolved analysis of border deflection as a proxy for traction forces at shared cell-cell interfaces. We also added a qualitative comparison of these tools to the Supplementary Text to better position our workflow relative to the current state of the art.*

*Changes to the text:*

*Introduction, page 5-6; line 107-113: “In addition, dedicated tools such as TISMorph, Junction Mapper and Polarity-JaM further provide morphometric and junction-related descriptors [30-32]. However, no single out-of-the-box workflow combines robust segmentation with exclusion of out-of-focus regions, quantification of tight-junction meandering, and in particular genotype-resolved analysis of border deflection as a proxy for traction forces at shared cell-cell interfaces. This highlights the need for a scalable workflow tailored to quantifying these junctional phenotypes.”*

*Results, page 7-8; line 149-162: “To assess whether established approaches are suitable for our ZO-1 imaging data and for tight-junction meandering quantification, we benchmarked CellProfiler, Cellpose-SAM, and Tissue Analyzer [28,29,33] on representative images and performed a qualitative comparision of the dedicated morphometric and junction-analysis tools TISMorph, Junction Mapper, and Polarity-JaM [30-32] (extensive Benchmarking in S1 Text). [...] A qualitative comparison with TISMorph, Junction Mapper, and Polarity-JaM showed that existing tools provide comparable junctional descriptors, but are not directly tailored to our specific readout.”*

*Discussion, page 15; line 348-357: “Quantitative analysis of epithelial morphology already builds on a substantial body of image-analysis methods. Established image-analysis platforms such as CellProfiler, Cellpose or Tissue Analyzer support segmentation, feature extraction, and epithelial morphometry [28,29,33,39], while dedicated tools such as TISMorph, Junction Mapper, and Polarity-JaM provide complementary descriptors of boundary irregularity, cell-cell contact organization, marker intensity, interface linearity and polarity [30-32]. These tools overlap conceptually with our analysis. However, our aim was not to replace existing platforms, but to derive assay-specific readouts for fine junctional meandering in ZO-1-stained monolayers and genotype-resolved behavior at shared borders in mixed-cell experiments, a combination not directly supported by the evaluated tools.”*

*S1 Text, page 2: “In addition to the segmentation-focused benchmarking, we performed a qualitative comparison of the dedicated morphometric and junction-analysis tools TISMorph [4], Junction Mapper [5], and Polarity-JaM [6] to assess segmentation strategy, descriptors for junctional morphology and support for genotype-resolved shared-border analysis in mixed-cell experiments.”*

*S1 Text, page 5-6: “Qualitative comparison with dedicated morphometric and junction-analysis tools:*

*To further position our workflow, we qualitatively compared it with existing tools for cell-shape and junction morphometry. This comparison focused on specific junctional parameters, segmentation strategies, and support for shared-border analysis in mixed-cell experiments.*

*TISMorph provides a broad set of single-cell morphology, texture, spreading, and boundary-irregularity descriptors, including measures related to boundary waviness and roughness. These features are conceptually related to our aim of quantifying contour irregularity. However, TISMorph relies on supervised threshold-based segmentation and CellProfiler-based cell detection, followed by analysis of individual cell objects. It is therefore well suited for detailed single-cell morphometry, but does not directly support automated extraction of shared epithelial borders or genotype-resolved analysis of heterotypic interfaces in mixed cultures.*

*Junction Mapper is specifically designed for cell-cell contact analysis and provides junction-level descriptors including interface length, marker intensity, fragmentation, and interface linearity. The interface linearity index is conceptually related to contour-based measures such as our R-index. Junction Mapper is particularly useful for intensity- and fragmentation-based junction phenotypes, including cases with discontinuous staining. However, it does not include task-specific deep learning segmentation for our ZO-1 image data and does not directly provide genotype-resolved directional shared-border analysis in mixed-cell experiments.*

*Polarity-JaM offers a broad feature set for polarity, morphology, marker intensity, flow, and junction analysis. Its segmentation is based on Cellpose, and its junction features build on Junction Mapper. While this makes Polarity-JaM a powerful exploratory framework, the Cellpose-based segmentation has the same limitations discussed in our benchmark for our ZO-1 dataset, particularly inclusion of out-of-focus regions and potential smoothing of fine junctional undulations. In addition, genotype-resolved mixed-culture border analysis is not available out of the box.”*

**Comment R1.1.3:** On the "deep learning" framing in the title and conclusion of the introduction (line 113): The use of "deep learning-supported" is appreciated and more accurate than "deep learning-based", as it correctly defines the DL component as assistive rather than foundational in the authors contribution. However, even this framing may still inadvertently draw the reader's attention toward the segmentation step rather than the actual methodological contribution. My suggestion would be to reorient the title around what the pipeline achieves rather than one of the tools it uses along the way. For example:

● "Semi-automated segmentation and morphometric quantification of epithelial tight junction organization in polycystic kidney disease"

● "A hybrid image analysis workflow for quantifying tight junction contour irregularities in polycystic kidney disease"

● "Morphometric characterization of epithelial tight junction irregularities using an automated segmentation and quantification pipeline in polycystic kidney disease"

This would more accurately show the novelty (the descriptor extraction and contour analysis) while the segmentation method (DL-supported or otherwise) becomes a means to that end.

***Response:*** *We thank the reviewer for the suggestions. We have considered the proposed alternatives, but prefer to retain the current title because it accurately reflects the workflow presented in this study and its deep learning-supported segmentation component.*

**Comment R1.1.4:** Also, I want to stress that I agree and endorse the modification that the authors did in the in line 162 in the subtitle “Tight junction meandering as a morphological phenotype”. It helped me to understand and introduce the section better.

***Response:*** *Thanks. No changes to the manuscript text.*

**Comment R1.2:** Reviewer reply: I found the new explanation in line 94 to 98. I understand now that it is an empirical measurement for the specific data of the authors as it is evaluated by members of the lab in the specific data. I thank the authors for the clarification.

***Response:*** *Thanks. No changes to the manuscript text.*

**Comment R1.3:** Reviewer reply: I would reword lines 474 and 475, CellProfiler and Tissue analyser don’t provide only “simple standard morphology measurements” for example Tissue Analyser extends CellProfiler features to epithelial tissue topology, quantifying cell-cell contact geometry, neighbourhood relationships, and junction network organisation directly from segmented monolayers.

***Response:*** *We thank the reviewer for pointing this out. This has been addressed in the revised Discussion, where we now describe CellProfiler, Cellpose, and Tissue Analyzer more broadly as platforms supporting segmentation, feature extraction, and epithelial morphometry, rather than limiting them to simple standard morphology measurements.*

*Discussion, page 15; line 348-357: “Quantitative analysis of epithelial morphology already builds on a substantial body of image-analysis methods. Established image-analysis platforms such as CellProfiler, Cellpose or Tissue Analyzer support segmentation, feature extraction, and epithelial morphometry [28,29,33,39], while dedicated tools such as TISMorph, Junction Mapper, and Polarity-JaM provide complementary descriptors of boundary irregularity, cell-cell contact organization, marker intensity, interface linearity and polarity [30-32]. These tools overlap conceptually with our analysis. However, our aim was not to replace existing platforms, but to derive assay-specific readouts for fine junctional meandering in ZO-1-stained monolayers and genotype-resolved behavior at shared borders in mixed-cell experiments, a combination not directly supported by the evaluated tools.”*

**Comment R1.4:** Reviewer reply: I appreciate the re-wording also in Figure7 supporting this section. That was helpful.

***Response:*** *Thanks. No changes to the manuscript text.*

**Comment R1.5:** Reviewer reply: “Some” users seems an understatement. I worry about the accessibility of this pipeline for the general bioimage analysis community who together are steering to use common standards (image formats, language and libraries) to be able to re-use the pipelines that other members develop.

***Response:*** *We thank the reviewer for raising this concern. As noted before, a full reimplementation is beyond the scope of this revision, but we improved reproducibility by providing the study datasets together with the existing code base.*

**Comment R1.6:** Reviewer reply: Thanks for the re-wording. I agree with the current state of the manuscript about the transferability and the re-wording of the Drosophila example.

***Response:*** *Thanks. No changes to the manuscript text.*

**Comment R1.7:** Reviewer reply: Excellent benchmark description, thanks to authors for putting the time on this to calculated times and quality.

***Response:*** *Thanks. No changes to the manuscript text.*

**Comment R1.8:** Reviewer reply: Thanks for the re-wording.

***Response:*** *Thanks. No changes to the manuscript text.*

**Comment R1.9:** Reviewer reply: As I mentioned before, I appreciate figure 7 and I think it mades the claim more understandable and supported. Great addition.

***Response:*** *Thanks. No changes to the manuscript text.*

**Comment R1.10:** Reviewer reply: Addressed and argued before. Advice is to change the title.

***Response:*** See response to R1.1.3.

**Comment R1.11:** Reviewer reply: Thanks for the clarification.

***Response:*** *Thanks. No changes to the manuscript text.*

**Comment R1.12:** Reviewer reply: Same as R1.11.

***Response:*** *Thanks. No changes to the manuscript text.*

**Comment R1.13:** Reviewer reply: The authors did a great work on their thorough discussion of segmentation methods, which genuinely strengthens the methodological section of the paper. However, this comment concerns a distinct and equally important aspect: the coverage of existing tools and workflows addressing morphological quantification of cell junctions and cell shape which is, after all, the core contribution of the paper. Several established tools that tackle quantification problems closely related to those addressed here are not cited or discussed. As noted in R1.1, Polarity-JaM is one example, but it is not the only one. Junction Mapper, and TISMorph, among others, offer overlapping or complementary morphometric descriptor sets including boundary irregularity, junction tortuosity, contour waviness, and shape complexity that are directly relevant to the claims made in this paper. The absence of these references makes it difficult to evaluate what the proposed workflow adds beyond what is already available, and where it genuinely has novelty. A comprehensive literature review of the morphological quantification landscape is necessary to correctly position the contribution, support claims about novelty, and ensure that the descriptors proposed are benchmarked or at least discussed in relation to existing ones. Without this, some of the novelty claims as currently stated risk being inaccurate or overstated, which in turn raises questions about the the conceptual framing of the paper.

***Response:*** *We thank the reviewer for this comment. As outlined in response to R1.1.2, we now discuss TISMorph, Junction Mapper, and Polarity-JaM in the main text and Supplementary Text to clarify the conceptual overlap with existing tools and to better define the assay-specific contribution of our workflow.*

**Comment R1.14:** Reviewer reply: Commented before, my concern about accessibility still stands.

***Response:*** *As noted previously, a full reimplementation is beyond the scope of this revision.*

**Comment R1.15:** I thank the authors for correcting all the minor observations and typos and I’d like to congratulate them for their efforts and their good work.

***Response:*** *We thank the reviewer for the positive assessment.*

**Reviewer #2:**

**Comment 2.1:** Abstract: Line 30: for clarity and completeness, I would also consider writing in full the abbreviation of “zonula occludens protein 1 (ZO1)” on its first appearance, just like you define ADPKD and MDCK. Also, for consistency, I would consider either ZO1 or ZO-1 and homogenise throughout the manuscript.

***Response:*** *We thank the reviewer for this comment. In the revised manuscript we now use ZO-1 throughout the manuscript and revised the abstract accordingly.*

*Abstract, page 2; line 30-31: “[...] immunofluorescence images of zonula occludens protein 1 (ZO-1)-stained monolayers.”*

**Comment 2.2:** Supplementary Material: S. Figure 3: typo in “Predefined”

***Response:*** *We thank the reviewer for pointing this out. This has been corrected.*

**Comment 2.3:** Supplementary Material: Text: In “and narrow limits of agreement (−1.085 to 1.371 μm²)”, I suspect these values refer to the Bland Altmann plots in SF5. Can you add a reference to the figure in the text to make it clearer, please? The negative values were unexpected at first.

***Response:*** *We thank the reviewer for this helpful suggestion. We added the figure references.*

*Supplementary Text S1, page 7: “For cell area, the correlation was near-perfect (Pearson r = 0.99995), with a minimal bias of 0.143 µm² and narrow limits of agreement (−1.085 to 1.371 µm²) (S5 Fig C). [...] Similarly, cell length showed extremely high agreement (r = 0.999741), with a negligible bias of −0.048 µm and tight limits of agreement (−0.228 to 0.132 µm) (S5 Fig D). [...] Cell width measurements were likewise highly consistent (r = 0.999606), with minimal bias (−0.016 µm) and narrow limits of agreement (−0.149 to 0.117 µm) (S5 Fig E).”*

**Comment 2.4:** Results: Line 183: “1388×1040 px; ~0.015 mm²” I would consider providing precise or estimates for both values consistently, unless 1388x1040 is fixed for all images. I would also replace “~” with “approximately” or equivalent.

***Response:*** *We thank the reviewer for this suggestion. We revised the sentence to clarify that the throughput estimate refers to the standard image size.*

*Results, page 9; line 188-190: “Our setup processed approximately 1,000 images per hour based on our standard image format of 1388 × 1040 pixels, corresponding to approximately 0.015 mm² per image, enabling high-throughput analysis.”*

**Comment 2.5:** Results: Line 208: I would consider rewriting “r_eq” in a formatted way without “_” and with eq as subscript. Same in Figure 6, 7 and their captions.

***Response:*** *We thank the reviewer for pointing this out. We have reformatted the affected variables using proper subscript notation throughout the figure captions.*

**Comment 2.6:** Discussion: Line 368: “in co-culture settings, it offers …” I believe the second comma is missing.

***Response:*** *We thank the reviewer for pointing this out. This has been corrected.*

*Discussion, page 16; line 378-389: “Nevertheless, in co-culture settings, it offers an additional layer of information that is [...].”*

**Comment 2.7:** Materials and Methods: Line 445: Is the w a typo? “stock w[118] was used…”.

***Response:*** *We thank the reviewer for pointing this out. We verified the stock designation and confirm that w[118] is correct; w refers to the white gene allele in Drosophila. Therefore, no change to the manuscript text was required.*

**Reviewer #3:**

Thank you for your revised manuscript, which made several things clearer.

**Comment 3.1:** General comments: Essentially, your requirement is to segment in-focus cell junctions while discarding out-of-focus ones. All the existing tools are developed for the segmentation of cells rather than tight junctions and therefore are developed to consider out of focus areas. Making this more explicit in the abstract and introduction would make the purpose of your work clearer. Since you later on also look at cell-specific measurements, this requirement gets lost in the remaining text. Streamlining your text with a clear focus would improve its readability.

***Response:*** *We thank the reviewer for this helpful comment. We have revised selected sentences in the Abstract, Introduction, and Results to make the central technical requirement clearer. We also clarified that downstream cell-level measurements are derived from this filtered tight-junction network.*

*Changes to the text:*

*Abstract, page 2; line 31-32: “Using a U-Net-based segmentation approach designed to mitigate out-of-focus regions, we [...].”*

*Introduction, page 5-6; line 107-113: “In addition, dedicated tools such as TISMorph, Junction Mapper and Polarity-JaM further provide morphometric and junction-related descriptors [30-32]. However, no single out-of-the-box workflow combines robust segmentation with exclusion of out-of-focus regions, quantification of tight-junction meandering, and in particular genotype-resolved analysis of border deflection as a proxy for traction forces at shared cell-cell interfaces. This highlights the need for a scalable workflow tailored to quantifying these junctional phenotypes.”*

*Results, page 8; line 168-172: “To address the limitations of conventional threshold-based segmentation methods, we trained a deep learning segmentation system using a U-Net convolutional network to detect ZO-1-stained tight junctions while ignoring out-of-focus regions unsuitable for downstream meandering analysis (S3 Fig).”*

*Results, page 9; line 195-196: “The filtered in-focus tight-junction network was then skeletonized to a [...].”*

**Comment 3.2:** The separation of the cell perimeter in the different branches of the tight junction network is a central point of your analysis. It should be illustrated in one of the figures.

***Response:*** *We thank the reviewer for this helpful suggestion. We agree that separation of the tight-junction network into individual border segments is central to the analysis. We therefore revised S6 Fig by adding panels illustrating the skeletonized ZO-1 junction network and its separation into individual border segments at branch points. The figure legend was updated accordingly.*

*Changes to the text:*

*Updated S6 Fig:*

*S6 Fig legend, page 30; line 753-756: “(C) Cropped region indicated in (B), showing the skeletonized ZO-1 junction network used for border extraction. (D) Separation of the skeletonized junction network into individual border segments. The network was split at branch points, and each border segment was assigned to the two adjacent cells and classified according to genotype pairing.”*

**Comment 3.3:** Specific comments: Line 177 “continued visual inspection over >2 years of workflow.” What is meant by that?

***Response:*** *We thank the reviewer for pointing this out. The statement referred to the fact that the workflow had been used over a period of more than two years without evidence of systematic segmentation errors during visual quality control, as described in the text. However, we agree that the wording was unclear and have removed this sentence from the revised manuscript.*

**Comment 3.4:** Line 196 “typical MSCK WT area …” How was this area measured? If you used the same pipeline for the measurement of the cell area, it would not be a valid argument.

***Response:*** *We thank the reviewer for this comment. The MDCK WT area was measured using the same analysis workflow after visual quality control of the detected cell objects. This value was not intended as an independent validation of the pipeline, but only to contextualize the predefined 25 µm² exclusion threshold, which is far below the typical MDCK WT cell size. In our view, this measurement is at least equivalent to manual area assessment, as manual tracing would use the same visually identified cell outlines, would likely be less precise, and would not be feasible at comparable scale. We therefore consider this cutoff appropriate for excluding very small fragmented detections or debris.*

**Comment 3.5:** Line 197 “subset of 12 images, … 1,173 cells” and Line 203 “5,477” Where these different subsets? How were they chosen? Why did you need different ones?

***Response:*** *We thank the reviewer for pointing out that this was not sufficiently clear. The two subsets were used for different purposes and were selected differently. The 12 images were chosen as representative examples to illustrate how many visually present cells are excluded by the predefined quality-control criteria and how many remain for downstream analysis. Because this required manual assessment of all cells in each image, this illustrative analysis was performed on a smaller image subset as part of the first revision. In contrast, the 5,477 cell objects were detected in randomly selected images and used to estimate the false detection rate among objects that had passed the automated detection and filtering steps. We have clarified this distinction in the revised manuscript.*

*Results, page 10; line 208-210: “Manual inspection of a larger separate randomly selected subset yielded a false detection rate (under- or over-segmented objects, as well as fused or split detections) of 0.4% (24/5,477)”*

**Comment 3.6:** Line 241 – 245 provide more details on the classification test. How many images? Ideally, show a confusion matrix.

***Response:*** *We thank the reviewer for this suggestion. We have revised the Results text to include the number of images used for the blinded classification test and added a confusion matrix to the Supplementary Information.*

*Changes to the text:*

*Results, page 11; line 248-251: “Immunofluorescence images of WT, PKD2⁻/⁻, and PKD2 rescue cells were assigned to genotype in a blinded manner based on junction appearance (riffled vs. intermediate vs. smooth). In total, 108 images were classified, and 80.6% were assigned correctly (87/108; confusion matrix in S1 Text).”*

*Supplementary S1 Text, page 7-8: Confusion matrix of blinded visual genotype classification based on tight-junction morphology*

| *True genotype* | *Classified as WT* | *Classified as PKD2 rescue* | *Classified as PKD2⁻/⁻* | *Total* |
| --- | --- | --- | --- | --- |
| *WT* | *28* | *8* | *0* | *36* |
| *PKD2 rescue* | *6* | *26* | *4* | *36* |
| *PKD2⁻/⁻* | *1* | *2* | *33* | *36* |
| *Total* | *35* | *36* | *37* | *108* |

*Rows indicate the true genotype after decoding; columns indicate the genotype assigned during blinded visual classification. Correctly classified: 87/108 = 80.6%. Misclassifications between WT and PKD2 rescue accounted for 14/21 incorrect assignments.”*

**Comment 3.7:** Line 272-274 You claim that you can use an area as an approximation for a force. Why is that valid? What are the assumptions that you have to make?

***Response:*** *We thank the reviewer for raising this important point. The original text already described the enclosed area as a geometric surrogate for junctional deformation rather than a direct force measurement. To make the underlying assumption more explicit, we revised the text to state that this interpretation assumes that directional border deflection reflects an imbalance in mechanical coupling across the shared interface and does not represent a direct force measurement.*

*Results, page 12; line 278-284: “The straight chord serves as a reference between endpoints while the enclosed area quantifies the deviation of the segmented border from this reference. Because this enclosed area corresponds to the integral of border displacement along the segment, it serves as a simple geometric surrogate for junctional deformation and, by extension, as a traction proxy for asymmetric force transmission along the shared border. This interpretation assumes that directional border deflection reflects an imbalance in mechanical coupling across the shared interface and does not represent a direct force measurement.”*

**Comment 3.8:** Legend of Fig 7 (B) “signed enclosed area” How do you decide on the sign of the area?

***Response:*** *We thank the reviewer for pointing this out. We clarified in the Fig 7 legend how the sign was assigned.*

*Results, Fig. 7 legend, page 13: “Traction proxy calculation: cytoskeletal traction at shared borders was estimated from the signed enclosed area of each border segment, with positive values predefined as deflection toward WT cells and negative values as deflection toward the opposing cell. For WT-WT borders, orientation was assigned randomly.”*

**Comment 3.9:** Line 318 -328, I do not think that you can make a conclusion about the performance of your pipeline for the Drosophila egg chamber from this one image. Undersegmentation also occurs for your own data set. You would need a systematic analysis of the percentage of undersegmentation in Drosophila. From my point of view, all you can state is that it is technically possible to feed this image into your pipeline.

***Response:*** *We thank the reviewer for this clarification. We agree that this analysis does not allow conclusions about segmentation performance or transferability to Drosophila epithelia. We therefore revised the section to describe it as a technical compatibility assessment only, stating that the images could be processed by the pipeline but that segmentation quality was insufficient for robust fully automated analysis without dataset-specific optimization. We state that reliable application to new cell types or imaging conditions would require fine-tuning using representative training images and systematic validation.*

*Results, page 14-15; paragraph 331-338: “To assess technical compatibility beyond MDCK monolayers, we applied the workflow to immunofluorescence images of Drosophila egg chamber epithelia stained for β-catenin, a marker of adherens junctions [32]. While these images could be processed by the pipeline, segmentation quality was not sufficient for robust fully automated analysis without dataset-specific optimization, and under-segmentation was observed in the representative example shown in S8 Fig. Reliable application to new cell types or imaging conditions would require additional fine-tuning using representative training images and systematic validation, which is expected to keep the required effort modest.”*
