# Supplementary material for: Deep learning-supported image quantification of epithelial cell shapes and its application to polycystic kidney disease
Source: PLoS Comput Biol. 2026 Aug 3;22(8):e1014614. doi: 10.1371/journal.pcbi.1014614 (PMC13456487; doi:10.1371/journal.pcbi.1014614)
Supplement: Supplementary file 1 [file pcbi.1014614.s011.pdf]

PLOS Computational article

Paper reviews

Deep learning-based image quantification of epithelial cell shapes and its application to polycystic kidney disease

Summary

The authors introduce a “deep-learning” based framework for shape quantification in epithelial cell and tight junction morphology from immunofluorescence images. They achieve that by building a pipeline that consists of a U-Net for the segmentation, followed by a pixel-based skeletonization step for the tight junctions. A second block for instance segmentation consists of watershed algorithms to obtain binary masks, followed by collection of measurements about the object such as area, perimeter and diameter. The authors also develop a model to quantify membrane meandering and riffled patterns comparing the perimeters of the original segmentation for each cell with a smoothed version using a novel metric proposed.

The main contributions of the paper are:

- A metric to quantify membrane meandering and riffled patterns is called “riffle-index (R-Index)”. The authors calculate that metric by comparing the smooth contour of each cell with the original segmentation silhouette. They conclude that cells with higher riffles exhibit larger perimeter differences. The author uses this difference to compute the R-index.
- An automated pipeline for epithelial cell shape quantification and tight junction morphology for immunofluorescence microscopy
- The framework build can generalize to other types of data with similar junctions.

Claims and evidences

The authors make several key claims, including:

Claim 1: A fully automated deep learning image analysis pipeline to quantify tight junction morphology in epithelial cells, with a focus on cell-based models for ADPKD based on empirical biological observations.
Claim 2: A novel metric for quantifying ADPKD phenotype and tight junction architecture has been developed and captures phenotypic differences in a manner consistent with manual assessment.
Claim 3: The authors claim that their automated approach clearly outperformed manual methods in terms of scalability, objectivity, and resolution of genotype-specific differences.

Claim 4: The authors implemented a traction model analyzing curvature at shared cell borders and its claim as a novelty because of the complexity of carrying them out manually.
Claim 5: The authors claim to demonstrate that a full end-to-end pipeline can be implemented using widely available tools such as ImageJ and Mathematica**.**
Claim 6: The authors claim that the method can generalise to other types of data without (drosophila example) without retrainning.

For some part, these claims are supported and explained by section "Results" in the papers and complemented by giving more details by "Materials and Methods" subsections such as "Training the deep learning network" and "Image analysis". However there are a couple of details to consider and addressed:

- The claim "*A fully automated deep learning image analysis pipeline to quantify tight junction morphology in epithelial cells, with a focus on cell-based models for ADPKD based on empirical biological observations.*" It's sufficiently described and supported by figure 2, figure 3, figure 4 and sections "Deep learning-based segmentation of ZO1 images", "Cell detection and unbiased measurements", "ADPKD modeling and riffle pattern quantification". However, there is one major point to address. The pipeline is presented as a "deep learning" pipeline and while it does have one step on the segmentation using Unet that is learning based approach I would advice the author to do not level all the method as "deep learning" specially because the novelties claimed a side of the pipeline itself are are happening outside the pipeline. Also, authors do not address SOTA in learning base approaches for Shape quantification in biology which I believe that if this is a paper about a novel deep learning approach it would be necessary to take the SOTA in the field in consideration.
- The claim "*A novel metric for quantifying ADPKD phenotype and tight junction architecture has been developed and captures phenotypic differences in a manner consistent with manual assessment*" is supported by the section "ADPKD modeling and riffle pattern quantification". The authors describe how they calculate the metric and argue why this metric is useful. However, the second part of the claim "*captures phenotypic differences in a manner consistent with manual assessment.*" I would like the authors to explain in detail how this comparison was made and if it was qualitative or quantitative in order to claim that they are consistent within each other.
- The claim "The authors claim that their automated approach clearly outperformed manual methods in terms of scalability, objectivity, and resolution of genotype-specific differences. " - it's not supported by quantitative results in any section. This is a big point in the author's discussion that they outperform other methods. The paper should qualitative and quantitatively describe how much and how this comparison has been carried out to support this claim.
- "Claim 4" is supported by the section "Results", subsection "Quantification of cytoskeletal traction between different phenotypes" and the panel C in the Fig 4 from the supplementary. The procedure is explained in an abstract manner however is sufficient to understand what the authors are calculating. Although, at the of the section the authors hint "This framework may enable detection of alterations in cytoskeletal force transmission between cells of different genotypes, which are difficult to assess manually." I'd love that the authors develop on this and show results that support this conclusion included in the supplementary or main paper.

- To the claim "*The authors claim to demonstrate that a full end-to-end pipeline can be implemented using widely available tools such as ImageJ and Mathematica.*". The author provides publicly available code in Github and this is a strong point for reproducibility. However, if the authors claim this to be a deep learning pipeline I strongly suggest turning this pipeline into Python language, more specifically Pytorch framework. Pytorch is the framework mostly used by bioimage analysis when it comes to deep learning development methods. Moreover, python is excellent and free and more accessible (as it's 100% free of licensing as opposed toMathematica and that makes it easier to be widely used by the community) library measurements such as scikit-image region props.
- And lastly, to the claim "The authors claim that the method can generalise to other types of data without (drosophila example) without retrainning." It is supported by the section "Results", subsection "Broader applications in cell biology" and figure 6. However, I would love that the authors do an actual qualitative comparison with another SOTA method such as CellProfiler without retraining. Ideally, take the same data and apply their pipeline.

Summary of changes, other comments and suggestions

Critical points to change or review

- I would encourage the authors to add more benchmarking to support their novel method and claims and make them stronger, some of them can be
    - Compare their Unet with some classical approach (non-learning) for instance Otsu threshold through Cellprofiler and calculate the time that each of them take and what is fast and how accurate because I have the intuition while the design of the UNet of the authors is absolutely valid and understandable, this approach maybe be long to train for the present data and authors will benefit more for a simpler and faster approach as they dataset is extensive and large. (e.i. Example -> CellProfiler modules to use: EnhanceEdges -> Threshold -> MorphologicalSkeleton). This is important also to show the relevance of the method VS current established methods otherwise its relevance may be questioned.
    - The paper should qualitative and quantitatively describe how much and how this comparison has been carried out to support this claim. Specially in claim 3 when authors add: "t their automated approach clearly outperformed manual methods in terms of scalability, objectivity, and resolution of genotype-specific differences"
- For claim 4, I'd love that the authors develop a figure and show results that support this conclusion included in the supplementary or main paper about "*This framework may enable detection of alterations in cytoskeletal force transmission between cells of different genotypes, which are difficult to assess manually*"
- The authors may reconsider to call this pipeline "Deep learning pipeline" for the reasons stated above.
- 133 and 134: "*We incorporated methods to ensure adequate segmentation and detection quality*" Which method did you implement? Please add a line clarifying that.

- 137 and 138: “*All algorithms were standardized to ensure robust, reproducible measurements across datasets.*” How were they standardised and why did it became more robust? I will add a sentence about this.
- More general points:
    - In general, I missed a bit of shape/morphology quantification methods in the SOTA. There are plenty of methods out there that can do feature extraction and it will be nice if the authors discuss them and why they are actually not an option for their analysis.
    - I would encourage the authors to consider move their code base to python.
    -

Minor details

- Fig 5, panel C, column 2: could the authors explain why “number of files” is relevant in this context? Otherwise I encourage you to remove this column.
- I saw that your documentation in Github is uploaded in .pdf format. I fully recommend to use .md (markdown format) as it is the standard recommended by the platform and takes less space than a .pdf when cloning the repo.
- In line 326: I would suggest to use the word “epochs” as opposed to iterations as it’s more frequently used in the Deep learning literature or when referring to training a method.
- 261: “Still, we recommend complementary manual inspection, particularly when applying the method to new cell types or imaging conditions.” - If that's the case I wouldn’t be comfortable to add the section of generalisation as this claim contradicts the fact that it’s generalisable when it comes for automatisation.
- In Figure 2 I wouldn't write any number as I think this is purely illustrative and it can divert the reader’s attention.
- Genotyp and widtyp typo should be Genotype and Wildtype in Figure 5

Questions for the authors

- In the lines 133-134 authors wrote: “*We incorporated methods to ensure adequate segmentation and detection quality, retaining only cells with fully detected ZO1 135 staining and no contact with the image border.*" Would you explain what those methods are? You give the error rate but don’t specify in the paper what those methods are to calculate the error.
- In line 188: the authors could explain with more detail about what quality control mean
- Question for the authors in line 181 “co-cultures containing GFP-labelled”, could you explain to me the value of co-cultures in that context?
- Lines 132 and 133: “*This approach converts binary segmentations into meaningful cell-level data, capturing full cell geometries rather than only border*.” Could the authors clarify what is the gain between only having the skeleton and a full field single color shape? I believe with the boundary (close curve only) you can calculate all the properties you enumerate in figure 3.

Conclusion

Overall, the core contributions are promising, and the method seems to potentially be able to help to achieve the goal of automated quantification of epithelial cell shape and tight junction morphology. I believe that it would be necessary to strengthen the paper with a more extensive SOTA review, more benchmarking to support the claims about the improved respect the current manual or classical approaches and some more the development in fundamental sections enumerated above in order to go ahead with the paper. I believe the approach used and the design pipeline make sense in order to achieve the author's purpose but more precise details of the methods used, quantitative analysis and plans to move their pipeline to Python would strengthen their paper. I thank the authors for their hard work on this paper and encourage them to keep working on it.
