# Supplementary material for: Deep learning-supported image quantification of epithelial cell shapes and its application to polycystic kidney disease
Source: PLoS Comput Biol. 2026 Aug 3;22(8):e1014614. doi: 10.1371/journal.pcbi.1014614 (PMC13456487; doi:10.1371/journal.pcbi.1014614)
Supplement: Supplementary file 2 [file pcbi.1014614.s012.pdf]

Manuscript – “Deep learning-based image quantification of epithelial cell shapes and its application to polycystic kidney disease”

Comments to authors:

The manuscript explores the use of deep-learning (DL) models to support the segmentation of epithelial cell contours from microscopy images with immunofluorescence staining and quantify their morphometry. Deep learning models for image analysis tasks have matured considerably over the last years, making established models promising for challenging segmentation tasks, such as the segmentation of cell contours presented here. This is a valuable application area where automation could significantly benefit the field. While the application of deep learning models is potentially valuable, I believe the manuscript requires improvements in problem contextualization within existing automated approaches, methodology description and comparison, and statistical rigor. Please find below some comments.

Introduction:

- Lines 72-73: “can provide valuable mechanistic insights.”: Can the authors please elaborate with specific examples of insights achieved with existing work and link it with the next sentence regarding the bottlenecks of such approaches? For example, in line 109, you write “To address the limitations of conventional threshold-based segmentation methods”, which you could already introduce here and highlight their limitations.
- Lines 77-78: “This facilitates automated and standardized quantification of cell shape across large datasets [17]”. Linked to the previous comment, this is a very plausible statement. Could the authors elaborate on existing attempts to use DL models in similar applications for cell morphology measurements and, if possible, any shortcomings?
- Lines 82-83: “introduce novel quantitative”: Can the authors please add a short statement as to why new quantitative features are needed? E.g. do they represent advances to previous metrics measured manually or entirely new parameters? Do they enable distinguishing groups of cells in a way that was previously not possible?
- Line 83: “tight junction configuration and cytoskeletal traction”: Could the authors add a brief statement about how this information is a reliable proxy to measure cell morphology information? (perhaps around lines 72-73).
- Line 84: “and reveals subtle morphogenetic differences”. It would strengthen the message to clarify if such differences are only “easily” detectable using this large-scale high-throughput pipeline (versus manual measurements).

Results:

- Lines 111-112: “Training images were randomly selected from diverse experiments to minimize selection bias and to improve generalizability.” It would be valuable to describe in more detail this selection in the methods. How many images were obtained from which experiments and any citations if available.
- Lines 115: “excluding out-of-focus regions”. How were these detected and handled? Is it still user dependent? The documentation mentions “Out of focus image parts might be ignored in the automatic processing.” but I believe it would be worth to already include a description of this step in the manuscript.
- Line 116: “evaluated by multiple researchers” Could you please specify the number?
- Lines 117-118: “accurate detection across varying background signals and artifacts, achieving an intersection over union of 0.771”: Given that the IoU can reach 1, it would be valuable to provide here or already in the introduction what is the baseline performance expected or to be achieved. Otherwise, it is unclear how or why you consider 0.771 an accurate detection.
- Lines 121: what is the typical evaluation time for manual analysis of such images? And what is the time-investment to prepare and annotate a dataset for this DL-based pipeline?
- Line 126: “unbiased measurements”: Could you be more specific, please?
- Lines 133-134: “We incorporated methods to ensure adequate segmentation and detection quality” Statements like this (or “followed a predefined strategy” in lines 114-115) come across as vague and would benefit from “See Methods ##” at the end to indicate where to find additional information, if you chose to not elaborate more in the main text. Do the authors refer to “general instructions provided by Falk et. al. [15]”? Could these instructions be briefly summarised (in either section).
- Line 136: is “n=5.477” the total number of cells? If yes, should “.” be removed since you also use the same symbol for decimal points?
- Line 137 “All algorithms were standardized”: After checking the methods section, I could not find what operations were considered for this purpose. Could the authors elaborate on these steps and justify how they do not hinder the detection of actual/true biological differences? (e.g. if one phenotype has consistently larger cell sizes than other but the measurements are standardised per dataset, such differences may become unclear.)
- Lines 148-149: “with a smoothed version of the original” is “surface” missing after “original”? My understanding from the figure is that the surface contour is smoothed. Can you clarify if the smoothing operation performed on an image with all cells, ensuring that the contour is the same regardless of the cell picked in the neighbourhood?
- Lines 162-163: “revealed strong concordance, with the automated analyses accurately reflecting visual assessments”: Can you clarify how was this assessed? Can it be measured? Otherwise it is again unclear what is the threshold for claiming “accuracy” between measurements. Also, can you elaborate how this measured when assessed manually?
- Lines 175: I find this section difficult to understand. Up until this point, it has become clear that cell borders are typically not smooth. Yet, it seems that for the cytoskeletal traction calculation, a

straight cell-boundary is assumed, which seems unrealistic. I also would think about traction being related to a displacement rather than an area. Could the authors justify this choice?

On another note, this section comes across as an implementation of a potentially valuable idea. However, statements like "This framework may enable detection" and the absence of a practical example showing how this metric can support quantitatively the analysis raises concerns. Could the authors highlight in their data a result that can only be obtained through this new metric? Additionally, I would expect the concept of cytoskeletal traction to be more emphasised in the introduction, strengthening the gap and need of new approaches to measure it.

- Line 194: it is a valuable effort to show applicability in a different dataset, as an example of transfer learning. Since the input images look very similar to previous datasets, there is an expectation that the same model should be competent in this application too. Do the authors have data to support "cell shape regulation" *in vivo* too? (similar to the comparison between 2 states: wild-type and PKD2 cells). In my opinion, that would strengthen and support the statement that the approach can also be used broadly for in vivo applications rather than a direct application for image processing.
- Lines 198: "achieved satisfactory performance", please provide values to specify what is considered acceptable.

Discussion:

- The authors picked one marker of cell junctions ZO-1. Can you include a comment on how transferable the approach is for other markers and cell types?
- Lines 219-231: This reads as an extended version of the similar paragraph from the introduction. I much prefer the version in the discussion for problem contextualisation and would favour it to appear in the introduction over the discussion.
- Lines 243: "clearly outperformed manual methods". As mentioned previously, there is an absence of metrics characterising existing manual approaches. It would strengthen the results to show how manual workflows compare quantitatively with the proposed pipeline (execution time, accuracy/errors…).
- Lines 261: "minimize general and systematic biases." A brief explanation of how you implemented this would be very helpful.

Materials and methods:

- A statistical analysis section should be added with the full analysis (e.g., tests to check normality of the data), followed by Mann-Whitney previously mentioned in the figure legend and any other tests performed.
- There are several instances mentioning that "out-of-focus regions were excluded". I could not find a description on how this process was carried out. Could the authors expand "Training the deep learning network" with how this was done, how many operators and how it influences the results?

- Line 319: Can the authors add details regarding train-validation-test splits of the dataset and potential cross-validation evaluation of the performance? Additionally, I would expect a baseline method to be presented (e.g. thresholding, briefly mentioned in the text), or manual annotation or a state-of-the-art model (like CellPose). Can the authors add such a comparison?
- Lines 320-321: "Image annotation was conducted in 3D Slicer" it would also be valuable to clarify how many operators performed annotations, their experience level and how these annotations were homogenised to define the ground truth / training data.

Figures:

- Figure 4: can cell #18 be added to A for completeness, so this reference doesn't appear unexpectedly in C. Also unclear why it is numbered with 18 if no other cells are labelled in the figure. Perhaps it could just be "Example" or similar.
- Figure 5: "Wildtype", typo? (or Wild-type to be consistent with other instances like in line 181) . Can you provide a measure of dispersion in table C? (Since you are reporting means, the standard deviation?)
- Figure 6: There are visible instances in B of cells that appear merged (either due to lack of staining or segmentation artefacts). How are these handled?
- Figure S4: Light-green regions should also be labelled. It is also unclear how the small gray areas inside green regions are handled. In B, how are there contours for cells which are nearly invisible in A?
- Figure S5: Is panel A part of panel B? If yes, can it be indicated in B?

Data and code availability:

The authors did a fantastic job with their documentation, and supporting screenshots. For the repository, I would consider adding the details regarding software requirements and versions already in the main Readme file.
