# Supplementary material for: Deep learning-supported image quantification of epithelial cell shapes and its application to polycystic kidney disease
Source: PLoS Comput Biol. 2026 Aug 3;22(8):e1014614. doi: 10.1371/journal.pcbi.1014614 (PMC13456487; doi:10.1371/journal.pcbi.1014614)
Supplement: Supplementary file 3 [file pcbi.1014614.s014.pdf]

Manuscript – "Deep learning-based image quantification of epithelial cell shapes and its application to polycystic kidney disease"

Comments to authors:

Thank you for the extensive replies to the comments raised previously, which have greatly improved my understanding of the work. I would have some additional minor topics to be clarified (please see below).

Abstract:

- Line 30: for clarity and completeness, I would also consider writing in full the abbreviation of "zonula occludens protein 1 (ZO1)" on its first appearance, just like you define ADPKD and MDCK. Also, for consistency, I would consider either ZO1 or ZO-1 and homogenise throughout the manuscript.

Supplementary Material:

- S. Figure 3: typo in "Predefined"
- Text:
  - In "and narrow limits of agreement (–1.085 to 1.371 μm^2)", I suspect these values refer to the Bland Altmann plots in SF5. Can you add a reference to the figure in the text to make it clearer, please? The negative values were unexpected at first.

Results:

- Line 183: "1388×1040 px; ~0.015 mm^2" I would consider providing precise or estimates for both values consistently, unless 1388x1040 is fixed for all images. I would also replace "~" with "approximately" or equivalent.
- Line 208: I would consider rewriting "r_eq" in a formatted way without "_" and with eq as subscript. Same in Figure 6, 7 and their captions.

Discussion:

- Line 368: "in co-culture settings, it offers …" I believe the second comma is missing.

Materials and Methods:

- Line 445: Is the w a typo? "stock w[118] was used…".
