# Supplementary material for: Deep learning-supported image quantification of epithelial cell shapes and its application to polycystic kidney disease
Source: PLoS Comput Biol. 2026 Aug 3;22(8):e1014614. doi: 10.1371/journal.pcbi.1014614 (PMC13456487; doi:10.1371/journal.pcbi.1014614)
Supplement: Supplementary file 4 [file pcbi.1014614.s015.pdf]

Review of the paper for PLOS
Deep learning-supported image quantification of epithelial cell shapes

Comments about the reviews and paper improvements for PLOS

Thanks to the authors for taking the time to reply and address all our concerns and all the hard work put in the paper.

R1.1: Reviewer reply: On the role of segmentation in quantification (line 104): The current phrasing implies that segmentation alone is necessary for quantification. It is worth noting that quantification does not always require segmentation (it is for shape quantification but not for image quantification); for instance, intensity-based or texture-based measurements can be performed directly on the raw image without prior segmentation.

On the claim of a lacking scalable workflow (line ~104): The statement "What remains lacking is a scalable workflow that preserves fine tight-junction contour irregularities and extracts descriptors tailored to this readout, including junction meandering and analysis of traction forces at cell-cell contacts" is not sufficiently supported and is directly challenged by existing tools such as Polarity-JaM (Nature Communications, 2025), which address different biological goals through similar approaches. The authors may legitimately argue that their method is simpler, faster, or more accurate for their specific use case (which I would absolutely agree with them) but claiming that such workflows are entirely lacking in the literature is an overstatement that a more comprehensive review of the current state of the art would correct.

On the "deep learning" framing in the title and conclusion of the introduction (line 113): The use of "deep learning-supported" is appreciated and more accurate than "deep learning-based", as it correctly defines the DL component as assistive rather than foundational in the authors contribution. However, even this framing may still inadvertently draw the reader's attention toward the segmentation step rather than the actual methodological contribution. My suggestion would be to reorient the title around what the pipeline achieves rather than one of the tools it uses along the way. For example:

- "Semi-automated segmentation and morphometric quantification of epithelial tight junction organization in polycystic kidney disease"
- "A hybrid image analysis workflow for quantifying tight junction contour irregularities in polycystic kidney disease"
- "Morphometric characterization of epithelial tight junction irregularities using an automated segmentation and quantification pipeline in polycystic kidney disease"

This would more accurately show the novelty (the descriptor extraction and contour analysis) while the segmentation method (DL-supported or otherwise) becomes a means to that end.

Also, I want to stress that I agree and endorse the modification that the authors did in the in line 162 in the subtitle “Tight junction meandering as a morphological phenotype”. It helped me to understand and introduce the section better.

R1.2. Reviewer reply: I found the new explanation in line 94 to 98. I understand now that it is an empirical measurement for the specific data of the authors as it is evaluated by members of the lab in the specific data. I thank the authors for the clarification.

R1.3. Reviewer reply: I would reword lines 474 and 475, CellProfiler and Tissue analyser don’t provide only “simple standard morphology measurements” for example Tissue Analyser extends CellProfiler features to epithelial tissue topology, quantifying cell-cell contact geometry, neighbourhood relationships, and junction network organisation directly from segmented monolayers.

R1.4. Reviewer reply: I appreciate the re-wording also in Figure7 supporting this section. That was helpful.

R1.5. Reviewer reply: “Some” users seems an understatement. I worry about the accessibility of this pipeline for the general bioimage analysis community who together are steering to use common standards (image formats, language and libraries) to be able to re-use the pipelines that other members develop.

R1.6. Reviewer reply: Thanks for the re-wording. I agree with the current state of the manuscript about the transferability and the re-wording of the Drosophila example.

R1.7. Reviewer reply: Excellent benchmark description, thanks to authors for putting the time on this to calculated times and quality.

R1.8. Reviewer reply: Thanks for the re-wording.

R1.9. Reviewer reply: As I mentioned before, I appreciate figure 7 and I think it mades the claim more understandable and supported. Great addition.

R1.10. Reviewer reply: Addressed and argued before. Advice is to change the title.

R1.11. Reviewer reply: Thanks for the clarification.

R1.12. Reviewer reply: Same as R1.11.

R1.13. Reviewer reply: The authors did a great work on their thorough discussion of segmentation methods, which genuinely strengthens the methodological section of the paper. However, this comment concerns a distinct and equally important aspect: the coverage of existing tools and workflows addressing morphological quantification of cell junctions and cell shape which is, after all, the core contribution of the paper. Several established tools that tackle quantification problems closely related to those addressed here are not cited or discussed. As noted in R1.1, Polarity-JaM is one example, but it is not the only one. Junction Mapper, and TISMorph, among others, offer overlapping or complementary morphometric descriptor sets including boundary irregularity, junction

tortuosity, contour waviness, and shape complexity that are directly relevant to the claims made in this paper. The absence of these references makes it difficult to evaluate what the proposed workflow adds beyond what is already available, and where it genuinely has novelty. A comprehensive literature review of the morphological quantification landscape is necessary to correctly position the contribution, support claims about novelty, and ensure that the descriptors proposed are benchmarked or at least discussed in relation to existing ones. Without this, some of the novelty claims as currently stated risk being inaccurate or overstated, which in turn raises questions about the the conceptual framing of the paper.

R1.14. Reviewer reply: Commented before, my concern about accessibility still stands.

I thank the authors for correcting all the minor observations and typos and I’d like to congratulate them for their efforts and their good work.
