# Supplementary material for: Deep learning-supported image quantification of epithelial cell shapes and its application to polycystic kidney disease
Source: PLoS Comput Biol. 2026 Aug 3;22(8):e1014614. doi: 10.1371/journal.pcbi.1014614 (PMC13456487; doi:10.1371/journal.pcbi.1014614)
Supplement: Supplementary file 5 [file pcbi.1014614.s016.pdf]

Response to Reviewers

We sincerely thank you for reviewing our manuscript titled "Deep learning-based image quantification of epithelial cell shapes and its application to polycystic kidney disease". We greatly appreciate your careful and constructive evaluation of our manuscript, which has greatly enhanced the clarity and quality of our work. Below, we provide detailed responses to each comment. Line numbers in the comments refer to the original submission unless noted otherwise.

Reviewer 1

R1.1. Comment: Claim 1 / general framing of the method as a "deep learning pipeline" and missing discussion of state-of-the-art learning-based approaches for morphology analysis.

Response: We agree that the original wording overstated the role of deep learning within the overall workflow. We therefore revised the manuscript to describe the method consistently as a deep learning-supported workflow rather than a purely deep learning pipeline. We also expanded the Introduction and Discussion to position the work relative to existing segmentation and morphology-analysis tools and to clarify that the key advance lies in assay-specific readouts of junctional meandering and shared-border behavior, not in generic segmentation alone.

R1.2. Comment: Claim 2: the manuscript states that the R-index captures phenotypic differences in a manner consistent with manual assessment, but the comparison was not explained in sufficient detail.

Response: We revised the Results section to describe the manual comparison more clearly. Manual assessment was performed as a blinded qualitative reference rather than as a formal quantitative benchmark. We now explain this explicitly, describe the comparison procedure in more detail, and avoid wording that could be interpreted as a direct quantitative equivalence between manual scoring and automated readouts.

R1.3. Comment: Claim 3: the statement that the automated approach "clearly outperformed manual methods" was not quantitatively supported.

Response: We agree and removed this statement. Because manual analysis in our study served only as a blinded qualitative reference, and not as a standardized time-resolved manual workflow, we do not believe that a formal benchmark of speed, error rates, or accuracy can be robustly derived from the present dataset. The revised Discussion therefore frames the strengths of the pipeline more cautiously in terms of scalability, standardization, and reduced user dependence.

R1.4. Comment: Claim 4: the traction framework was potentially interesting, but the manuscript did not provide enough concrete evidence for its value.

Response: We revised the traction section substantially and added a more explicit practical example in the co-culture setting. The revised text now explains more clearly what is computed, why it is biologically relevant, and how the readout can reveal genotype-dependent differences at shared borders that are difficult to capture reproducibly by visual inspection alone.

R1.5. Comment: Claim 5: the end-to-end pipeline is implemented in ImageJ and Mathematica; the reviewer suggested a Python/PyTorch implementation.

Response: We agree that a Python implementation would improve accessibility for some users. However, a full reimplementation is beyond the scope of the present revision. To maximize reproducibility in the current framework, we provide detailed documentation and sample data in the repository, which should provide a practical starting point for users who wish to apply the workflow to their own datasets or build related analysis pipelines.

R1.6. Comment: Claim 6: the manuscript implied that the method generalized to other datasets without retraining.

Response: We agree that the original generalization claim was too strong. We therefore revised the relevant Results and Discussion sections to describe the Drosophila example more narrowly as a proof-of-principle transfer to a related epithelial dataset. We now state explicitly that robust transfer to new markers or tissues will require dataset-specific retraining or validation.

R1.7. Comment: Compare their Unet with some classical approach (non-learning) for instance Otsu threshold through Cellprofiler and calculate the time that each of them take and what is fast and how accurate because I have the intuition while the design of the UNet of the authors is absolutely valid and understandable, this approach maybe be long to train for the present data and authors will benefit more for a simpler and faster approach as they dataset is extensive and large. (e.i. Example -> CellProfiler modules to use: EnhanceEdges -> Threshold -> MorphologicalSkeleton). This is important also to show the relevance of the method VS current established methods otherwise its relevance may be questioned.

Response: In response, we added a focused benchmark against established tools, including CellProfiler, Cellpose-SAM, and Tissue Analyzer. This comparison is now summarized in the Results and described in more detail in the supplementary S1 Text. We also added a figure showing results from each pipeline (S9 Fig). The revised text emphasizes that while existing tools provide strong general segmentation and morphometry, they did not directly support the assay-specific combination of out-of-focus-aware analysis, meandering quantification, and genotype-resolved shared-border analysis required for our workflow.

R1.8. Comment: The paper should qualitative and quantitatively describe how much and how this comparison has been carried out to support this claim. Specially in claim 3 when authors add: "their automated approach clearly outperformed manual methods in terms of scalability, objectivity, and resolution of genotype-specific differences".

Response: We addressed this by removing the unsupported superiority claim and by clarifying the role of manual assessment as a blinded qualitative reference. We also note in the revised text that earlier studies have typically relied on small-scale, labor-intensive manual quantification of selected junction segments, rather than on an established gold-standard manual workflow that could serve as a formal benchmark.

R1.9. Comment: For claim 4, I'd love that the authors develop a figure and show results that support this conclusion included in the supplementary or main paper about "This framework may enable detection of alterations in cytoskeletal force transmission between cells of different genotypes, which are difficult to assess manually".

Response: We thank the reviewer for this helpful suggestion. In response, we added results from a mixed-culture experiment to directly illustrate this point. The new Fig. 7 now shows both meandering quantification at shared borders and the corresponding traction-proxy analysis. These data provide a

concrete example of how the framework can resolve genotype-dependent behavior at heterotypic interfaces and thereby reveal differences in intercellular force transmission that would be difficult to assess reliably by manual inspection alone.

R1.10. Comment: The authors may reconsider to call this pipeline "Deep learning pipeline" for the reasons stated above.

Response: We thank the reviewer for this helpful point. Throughout the revised manuscript, we now refer to the method more precisely as a deep learning-supported workflow or deep learning-supported image-analysis pipeline.

R1.11. Comment: "We incorporated methods to ensure adequate segmentation and detection quality" Which method did you implement? Please add a line clarifying that.

Response: We thank the reviewer for pointing that out. To address this point, we revised the corresponding Methods and Results passages to describe the implemented quality-control strategy more explicitly. In brief, uniform predefined filters were applied across all datasets and genotypes to exclude border-touching objects, objects lacking a closed ZO1 contour, merged-cell candidates, and very small objects. After these fixed criteria were applied, all remaining objects were included without further dataset- or phenotype-specific selection. We now also report the corresponding manual quality check and false detection rate in the revised manuscript (Revised line 182-200).

R1.12. Comment: "All algorithms were standardized to ensure robust, reproducible measurements across datasets." How were they standardised and why did it became more robust? I will add a sentence about this.

Response: We understand the reviewer's concern that the original wording was too general and did not sufficiently explain what was meant by standardization or why this would improve robustness. We therefore revised this section substantially and replaced the broad statement with a more explicit description of the standardized processing steps. As described in comment R1.11 the revised manuscript now states more concretely which fixed processing steps and exclusion criteria were applied uniformly (Revised line 182-200).

R1.13. Comment: In general, I missed a bit of shape/morphology quantification methods in the SOTA. There are plenty of methods out there that can do feature extraction and it will be nice if the authors discuss them and why they are actually not an option for their analysis.

Response: We thank the reviewer for this important point. In response, we substantially expanded the discussion of the state of the art in morphology and shape quantification, both in the main text and in the Supplementary Material. Specifically, we added discussion of previously used manual approaches, including zig-zag- and tortuosity-based measures, and positioned our workflow more clearly relative to established image-analysis platforms such as CellProfiler, Cellpose-SAM, and Tissue Analyzer. To address this more directly, we also included a focused benchmark against these tools. Because our aim was not generic cell segmentation alone, but reliable quantification of tight-junction meandering under heterogeneous imaging conditions, we specifically evaluated preservation of fine junctional undulations and false inclusion of out-of-focus regions. In a manually reviewed subset of 12 representative images, our U-Net pipeline showed the lowest false-inclusion rate (5.0%), compared with CellProfiler (13.7%) and Tissue Analyzer (34.0%). We therefore revised the manuscript to present our workflow more clearly as a task-specific solution for tight-junction phenotyping and downstream meandering analysis, rather than as a general replacement for existing morphology tools.

R1.14. Comment: I would encourage the authors to consider move their code base to python.

Response: We thank the reviewer for this helpful suggestion. We agree that a Python implementation would improve accessibility for some users. However, a full migration of the code base is beyond the scope of the present revision. To improve reproducibility within the current framework, we added the datasets used in this study to the repository together, so that the existing implementation can be reproduced more easily and can serve as a practical starting point for users who wish to adapt or extend the analysis.

R1.15. Minor details: Fig 5, panel C, column 2: could the authors explain why "number of files" is relevant in this context? Otherwise I encourage you to remove this column.

Response: We retained the "number of files" column because statistical aggregation in this analysis was performed at the file level rather than at the level of individual cells, in order to avoid pseudoreplication. The number of files therefore indicates the effective number of independent measurement units contributing to the statistical comparison. We have revised the figure legend/text to clarify this rationale more explicitly (Revised Fig 6).

R1.16. Minor details: I saw that your documentation in Github is uploaded in .pdf format. I fully recommend to use .md (markdown format) as it is the standard recommended by the platform and takes less space than a .pdf when cloning the repo.

Response: Done. The repository documentation was revised and moved into a markdown file.

R1.17. Minor details: In line 326: I would suggest to use the word "epochs" as opposed to iterations as it's more frequently used in the Deep learning literature or when referring to training a method.

Response: Done. The terminology was revised accordingly (Revised line 484).

R1.18. Minor details: 261: "Still, we recommend complementary manual inspection, particularly when applying the method to new cell types or imaging conditions." - If that's the case I wouldn't be comfortable to add the section of generalisation as this claim contradicts the fact that it's generalisable when it comes for automatization.

Response: We agree with the reviewer that the original wording overstated the degree of generalizability. In the revised manuscript, we therefore removed the stronger claim of direct generalizability and reframed this part as a proof-of-principle transfer to a related epithelial imaging dataset. We now state more explicitly that application to new cell types or imaging conditions may require dataset-specific fine-tuning/retraining to ensure robust segmentation quality. At the same time, we emphasize that such adaptation is feasible with a limited number of representative training images, which keeps the required effort manageable. We believe this revised framing more accurately reflects the capabilities and limitations of the workflow.

R1.19. Minor details: In Figure 2 I wouldn't write any number as I think this is purely illustrative and it can divert the reader's attention.

Response: Done. The figure was simplified accordingly. (Revised Fig 3)

R1.20. Minor details: Genotyp and widtyp typo should be Genotype and Wildtype in Figure 5

Response: Done. These typographical errors were corrected.

R1.21. Questions: In the lines 133-134 authors wrote: "We incorporated methods to ensure adequate segmentation and detection quality, retaining only cells with fully detected ZO1 135 staining and no contact with the image border." Would you explain what those methods are? You give the error rate but don't specify in the paper what those methods are to calculate the error.

Response: We thank the reviewer for this important question. We revised the relevant Results sections to describe the quality-control workflow more explicitly. The revised manuscript now states that uniform predefined filters were applied across all datasets and genotypes, including exclusion of border-touching objects, objects lacking a closed ZO1 contour, merged-cell candidates, and very small objects. We also now quantify how many objects were excluded by these criteria in a manually reviewed representative subset and report the resulting false detection rate after filtering. Because additional biological replicates and image fields can be generated relatively easily in this system, we chose a deliberately conservative filtering strategy in order to avoid including incorrectly segmented cells in the downstream analysis. This was intended to prioritize detection quality over maximal cell yield. (Revised line 182-200)

R1.22. Questions: In line 188: the authors could explain with more detail about what quality control mean.

Response: We clarified that quality control refers to a short post-analysis review to detect gross systematic failures of segmentation or unsuitable preparations, rather than manual curation of individual cells. This distinction is now stated more explicitly. (Revised line 299-301)

R1.23. Questions: Question for the authors in line 181 "co-cultures containing GFP-labelled", could you explain to me the value of co-cultures in that context?

Response: We thank the reviewer for this important question. We revised the co-culture section to explain the rationale more clearly. The value of the co-culture setup is that it allows cells of different genotypes to be analyzed within the same epithelial field and under identical staining, imaging, and culture conditions. GFP labeling enables unambiguous genotype assignment of neighboring cells and therefore makes it possible to distinguish homotypic from heterotypic shared borders within one monolayer. This is particularly useful for the present analysis because the traction proxy and shared-border meandering are intended to capture genotype-dependent behavior at cell–cell interfaces. In a mixed culture, such effects can be assessed directly at borders between different cell types, rather than being inferred indirectly from separate monocultures. The co-culture design therefore provides a more stringent setting to test whether the workflow can detect interface-specific differences in junctional organization and intercellular mechanical coupling.

R1.24. Questions: Lines 132 and 133: "This approach converts binary segmentations into meaningful cell-level data, capturing full cell geometries rather than only border." Could the authors clarify what is the gain between only having the skeleton and a full field single color shape? I believe with the boundary (close curve only) you can calculate all the properties you enumerate in figure 3.

Response: We agree that this wording was confusing. Since the intended distinction was more biological than technical, we removed the sentence rather than defending an imprecise formulation.

Reviewer 2

R2.1. Comment Introduction: Lines 72-73: "can provide valuable mechanistic insights.": Can the authors please elaborate with specific examples of insights achieved with existing work and link it with the next sentence regarding the bottlenecks of such approaches? For example, in line 109, you write "To address the limitations of conventional threshold-based segmentation methods", which you could already introduce here and highlight their limitations.

Response: We thank the reviewer for this helpful suggestion. We revised the Introduction to provide more specific examples of mechanistic insights obtained from previous work. In particular, we now refer to studies in which manual quantification of tight-junction tortuosity, ruffling, or zig-zag morphology was used to relate junctional architecture to epithelial remodeling and cytoskeletal organization. We also now explicitly link these examples to the limitations of such approaches by stating that these analyses are typically time-consuming, user-dependent, and usually restricted to relatively small numbers of manually selected bicellular junction segments rather than entire monolayers. This revision was intended to clarify both the value of existing approaches and the bottlenecks that motivated the present study. (Revised line 96-101)

R2.2. Comment Introduction: Lines 77-78: "This facilitates automated and standardized quantification of cell shape across large datasets [17]". Linked to the previous comment, this is a very plausible statement. Could the authors elaborate on existing attempts to use DL models in similar applications for cell morphology measurements and, if possible, any shortcomings?

Response: We agree and have revised the Introduction accordingly. We now acknowledge that deep learning-based segmentation has substantially advanced biomedical image analysis and has been incorporated into general image-analysis tools used for cell segmentation and epithelial morphometry. At the same time, we now clarify more explicitly that segmentation alone does not solve the assay-specific quantification problem addressed here. Specifically, existing general-purpose segmentation pipelines are not designed to quantify fine tight-junction contour irregularities, junction meandering, or genotype-resolved shared-border phenotypes in mixed-cell experiments. We therefore distinguish more clearly between advances in segmentation and the remaining gap in readout-specific quantitative analysis. (Revised line 143-155)

R2.3. Comment Introduction: Lines 82-83: "introduce novel quantitative": Can the authors please add a short statement as to why new quantitative features are needed? E.g. do they represent advances to previous metrics measured manually or entirely new parameters? Do they enable distinguishing groups of cells in a way that was previously not possible?

Response: Thank you for this important point. We have now added an explicit statement to address this issue. The revised Introduction now states: "New quantitative features are therefore needed to move beyond manually derived low-dimensional metrics and to capture junctional phenotypes in a scalable and standardized manner across large image datasets." (Revised line 110-112) This was added to clarify that the motivation is not only automation per se, but also the need to extend beyond manually derived measurements such as tortuosity or zig-zag scoring toward a more systematic and scalable characterization of epithelial junction phenotypes. Also, genotype-resolved shared-border analysis is only partly feasible by visual inspection.

R2.4. Comment Introduction: Line 83: "tight junction configuration and cytoskeletal traction": Could the authors add a brief statement about how this information is a reliable proxy to measure cell morphology information? (perhaps around lines 72-73).

Response: We thank the reviewer for this helpful suggestion. We have revised the Introduction, Results and Discussion to clarify the biological rationale for these readouts. Specifically, we now state that tight-junction configuration can serve as a proxy for changes in cortical mechanics and junction–cytoskeleton coupling, and that border-associated cytoskeletal traction provides a complementary readout of intercellular force transmission. This addition was intended to explain more explicitly why these measurements are informative proxies for morphologically relevant differences in epithelial organization and mechanics. (Revised line 259-269; 356-362)

R2.5. Comment Introduction: Line 84: “and reveals subtle morphogenetic differences”. It would strengthen the message to clarify if such differences are only “easily” detectable using this large-scale high-throughput pipeline (versus manual measurements).

Response: We agree and have revised the relevant section to make this point clearer. In the Introduction, we now emphasize that manual quantification approaches are low-throughput, user-dependent, and typically restricted to selected junction segments, whereas the workflow presented here enables standardized high-throughput analysis across large image datasets. Our intention was to clarify that subtle morphogenetic differences are more readily and reproducibly detected in this large-scale, standardized framework than by manual inspection or limited low-throughput quantification alone. (Revised line 96-103; S2 Sig)

R2.6. Comment Results: Lines 111-112: “Training images were randomly selected from diverse experiments to minimize selection bias and to improve generalizability.” It would be valuable to describe in more detail this selection in the methods. How many images were obtained from which experiments and any citations if available.

Response: The training subsection in the Methods was expanded substantially. It now states how the images were selected, what kinds of variation were represented, and how the train/validation/test sets were defined. (Revised line 451-492)

R2.7. Comment Results: Lines 115: “excluding out-of-focus regions”. How were these detected and handled? Is it still user dependent? The documentation mentions “Out of focus image parts might be ignored in the automatic processing.” but I believe it would be worth to already include a description of this step in the manuscript.

Response: We thank the reviewer for this important point. We have added this information to both the annotation/training description and the Results. In brief, out-of-focus regions were identified manually during annotation based on predefined morphological criteria, including (i) an apparent widening of the tight-junction signal by more than 100% compared with adjacent in-focus regions and/or (ii) a clear straightening or loss of the characteristic meandering border appearance. Such regions, together with background noise and non-junctional signal, were annotated as negative class and were therefore explicitly included during training. This strategy was intended to reduce interference from blurred structures in downstream segmentation and morphometric analysis. Although the initial annotation step was manual, it followed standardized criteria and was cross-checked by two readers, so that user dependence was reduced as much as feasible within the annotation process. (Revised line 452-465)

R2.8. Comment Results: Line 116: “evaluated by multiple researchers” Could you please specify the number?

Response: Done. The number of researchers is now specified in the revised manuscript (n = 9). (Revised line 169)

R2.9. Comment Results: Lines 117-118: “accurate detection across varying background signals and artifacts, achieving an intersection over union of 0.771”: Given that the IoU can reach 1, it would be valuable to provide here or already in the introduction what is the baseline performance expected or to be achieved. Otherwise, it is unclear how or why you consider 0.771 an accurate detection.

Response: We rewrote this passage to explain that the target structure is a thin linear junction signal only a few pixels wide, so small boundary deviations disproportionately reduce overlap-based

metrics such as IoU. We also now note that the downstream morphometric consequences of these small deviations were limited. (Revised line 162-168)

R2.10. Comment Results: Lines 121: what is the typical evaluation time for manual analysis of such images? And what is the time-investment to prepare and annotate a dataset for this DL-based pipeline?

Response: We thank the reviewer for raising this point. In our hands, a purely qualitative, blinded manual assessment of a typical dataset of ~100 images—i.e., assigning each image to broad phenotype categories such as low/intermediate/high membrane ruffling—required approximately 1 hour. This approach, however, yields only a coarse categorical classification and does not provide object-based quantitative measurements at the level of individual cell boundaries. In contrast, establishing the deep-learning (DL) pipeline required a one-time annotation effort to generate training data. Manual labeling required approximately 1–2 hours per image, corresponding to ~20–25 hours for the complete training set used in this study. Once trained, the pipeline enabled standardized batch processing with quantitative readouts per cell object, which would not be feasible by manual inspection at comparable scale and consistency. We further note that true manual quantification of junctional "meandering" or zig-zag morphology at the level of individual cell borders (as performed in prior work; doi:10.1371/journal.pone.0104994) would require substantially greater time investment than the coarse image-level scoring described above and is therefore practical only for limited numbers of cells. We have clarified this distinction in the revised manuscript. (Revised line 303-308)

R2.11. Comment Results: Line 126: "unbiased measurements": Could you be more specific, please?

Response: We agreed that the wording was misleading and removed it. The revised manuscript now clearly states what steps were applied for standardization. (Revised line 182-200)

R2.12. Comment Results: Lines 133-134: "We incorporated methods to ensure adequate segmentation and detection quality" Statements like this (or "followed a predefined strategy" in lines 114-115) come across as vague and would benefit from "See Methods ##" at the end to indicate where to find additional information, if you chose to not elaborate more in the main text. Do the authors refer to "general instructions provided by Falk et. al. [15]"? Could these instructions be briefly summarised (in either section).

Response: We rewrote this entire part of the manuscript. The revised text now gives a more explicit description of the training strategy, object exclusion criteria, and quality-control steps, making the relevant procedures easier to follow. (Revised line 451-492)

R2.13. Comment Results: Line 136: is "n=5.477" the total number of cells? If yes, should "." be removed since you also use the same symbol for decimal points?

Response: Done. Number formatting was corrected for consistency. (Revised line 198)

R2.14. Comment Results: Line 137 "All algorithms were standardized": After checking the methods section, I could not find what operations were considered for this purpose. Could the authors elaborate on these steps and justify how they do not hinder the detection of actual/true biological differences? (e.g. if one phenotype has consistently larger cell sizes than other but the measurements are standardised per dataset, such differences may become unclear.)

Response: We agree with the reviewer that the original statement "All algorithms were standardized" was too unspecific and could be misunderstood as implying phenotype-dependent

normalization. We have therefore replaced this wording and now explicitly describe which fixed quality-control (QC) and exclusion steps were applied identically to all datasets and genotypes. Importantly, these steps do not normalize biological measurements (e.g., cell size, perimeter, or junctional morphology) within a dataset. Instead, they remove objects that cannot be reliably quantified due to technical artifacts (e.g., truncation at image borders, non-closed junction contours, merged-cell candidates, or defocus/segmentation failures). All remaining objects are subsequently quantified in physical units using the microscope pixel size, without dataset- or genotype-specific scaling, thereby preserving true biological differences. (Revised line 182-200)

R2.15. Comment Results: Lines 148-149: "with a smoothed version of the original" is "surface" missing after "original"? My understanding from the figure is that the surface contour is smoothed. Can you clarify if the smoothing operation performed on an image with all cells, ensuring that the contour is the same regardless of the cell picked in the neighbourhood?

Response: We clarified that smoothing is applied to the whole skeleton derived from the original image and added an additional Figure to show the effect (S5 Fig). We also now note explicitly that the same smoothing settings were used across all conditions. Also, we added a statistic analysis assessing the effect on object size, which shows no relevant differences on the overall cell morphology besides the riffle pattern through the smoothing procedure.

R2.16. Comment Results: Lines 162-163: "revealed strong concordance, with the automated analyses accurately reflecting visual assessments": Can you clarify how was this assessed? Can it be measured? Otherwise it is again unclear what is the threshold for claiming "accuracy" between measurements. Also, can you elaborate how this measured when assessed manually?

Response: We rewrote this part of the Results to explain the blinded visual assessment more clearly and to avoid overstating the comparison. The revised manuscript now makes clearer that this comparison served as a qualitative reference rather than a formal quantitative benchmark. We agree, that the initial wording was misleading and removed it. (Revised line 226-232)

R2.17. Comment Results: Lines 175: I find this section difficult to understand. Up until this point, it has become clear that cell borders are typically not smooth. Yet, it seems that for the cytoskeletal traction calculation, a straight cell-boundary is assumed, which seems unrealistic. I also would think about traction being related to a displacement rather than an area. Could the authors justify this choice? On another note, this section comes across as an implementation of a potentially valuable idea. However, statements like "This framework may enable detection" and the absence of a practical example showing how this metric can support quantitatively the analysis raises concerns. Could the authors highlight in their data a result that can only be obtained through this new metric? Additionally, I would expect the concept of cytoskeletal traction to be more emphasised in the introduction, strengthening the gap and need of new approaches to measure it.

Response: We thank the reviewer for this feedback and agree that the previous description was unclear. We revised the manuscript to clarify the concept, avoid overinterpretation as a physical traction measurement, and add a concrete data example (Fig 7).

First, our approach does not assume smooth or straight cell borders. The segmented ZO-1 contour is retained as a non-linear polyline. The straight chord is used only as a reference baseline connecting the same endpoints of a border segment, enabling quantification of lateral deflection of the observed contour relative to this baseline (S6 Fig C).

Second, we agree that traction is classically defined in terms of force and displacement. Accordingly, we now describe this measure as a geometric proxy for junctional deformation rather than a direct

traction readout. We use the enclosed area between the segmented border and the chord because it provides a robust scalar summary of border deflection; as stated in the manuscript, it corresponds to the integral of lateral displacement along the segment. Using the signed enclosed area further enables directional analysis at heterotypic borders.

Third, we added a practical example using WT.GFP–*Cgn*$^{-}$/$^{-}$ co-cultures, which generate homotypic and heterotypic borders within the same epithelial field under identical imaging conditions (Fig 7A, B). The R-index differed systematically by border pairing (*CGN*–*CGN* < *CGN*–WT < WT–WT; Fig 7C). Importantly, the enclosed-area proxy revealed a directional bias at heterotypic interfaces: WT–CNG borders shifted significantly toward WT, whereas WT–WT borders showed no directional bias (Fig 7D). This directional effect is difficult to quantify reliably by blinded visual inspection alone and illustrates the added value of the proposed border-level metric.

R2.18. Comment Results: Line 194: it is a valuable effort to show applicability in a different dataset, as an example of transfer learning. Since the input images look very similar to previous datasets, there is an expectation that the same model should be competent in this application too. Do the authors have data to support "cell shape regulation" in vivo too? (similar to the comparison between 2 states: wild-type and PKD2 cells). In my opinion, that would strengthen and support the statement that the approach can also be used broadly for in vivo applications rather than a direct application for image processing.

<u>Response:</u> We appreciate this important comment and agree that our original wording overstated the biological conclusion that can be drawn from the Drosophila example. This dataset was included to demonstrate portability of the image-analysis workflow to a related epithelial imaging context, not to establish cell-shape regulation in vivo with a genotype/state comparison analogous to our MDCK WT vs PKD2 analysis. Accordingly, we revised the manuscript to remove or soften statements implying a validated functional interpretation in vivo and to frame this section explicitly as an image-analysis transfer/portability experiment.

We also agree that, because the input images are visually similar to our in vitro datasets, it is reasonable to expect that the same model should perform competently; we therefore adjusted the text to emphasize feasibility of applying/adapting the existing model and pipeline rather than presenting this as evidence of a distinct biological finding. Finally, to provide appropriate biological context without overclaiming, we now cite prior work reporting related epithelial shape/junctional phenotypes in vivo (Martin et al., 2009), suggesting that the pipeline could potentially be applied to in vivo systems.

R2.19. Comment Results: Lines 198: "achieved satisfactory performance", please provide values to specify what is considered acceptable.

<u>Response:</u> We agree that the phrase "achieved satisfactory performance" was too subjective without accompanying quantitative benchmarking. Because the Drosophila example was based on a small external dataset and did not provide a sufficiently robust basis for quantitative performance assessment across multiple images, we chose not to add a weak or potentially misleading metric. Instead, we revised the text to avoid overinterpretation and now describe the result more cautiously as a qualitative proof of principle, while explicitly noting that segmentation quality was not yet sufficient for robust automated analysis without additional fine-tuning. (Revised line 313-322)

R2.20. Comment Discussion: The authors picked one marker of cell junctions ZO-1. Can you include a comment on how transferable the approach is for other markers and cell types?

Response: We thank the reviewer for this suggestion. We have added a brief discussion of transferability to the revised Discussion. Specifically, we now note that similar junctional phenotypes have been described with other markers, including ZO-2/ZO-3, actin, cingulin, and E-cadherin, and that the general analytical framework may therefore be extendable to related junctional readouts and epithelial systems. At the same time, we clarify that transfer will depend on image quality, marker continuity, staining characteristics, and dataset-specific retraining or validation. We therefore present the method as a marker- and assay-specific framework with extensibility, rather than as a universally transferable solution. (Revised line 369-381)

R2.21. Comment Discussion: Lines 219-231: This reads as an extended version of the similar paragraph from the introduction. I much prefer the version in the discussion for problem contextualisation and would favour it to appear in the introduction over the discussion.

Response: We agree and revised the structure accordingly. The broader contextualization of manual versus automated image analysis was moved to the Introduction, while the Discussion now focuses more directly on interpretation, value, limitations, and transferability

R2.22. Comment Discussion: Lines 243: "clearly outperformed manual methods". As mentioned previously, there is an absence of metrics characterising existing manual approaches. It would strengthen the results to show how manual workflows compare quantitatively with the proposed pipeline (execution time, accuracy/errors…).

Response: We thank the reviewer for this comment. We agree that, without a quantitative benchmark against a standardized manual workflow, the statement that the automated pipeline "clearly outperformed manual methods" was too strong. Because the manual analysis in our study served as a blinded qualitative reference rather than as a formal benchmark, we removed this wording and revised the Discussion accordingly. The revised text now frames the automated workflow more cautiously in terms of scalability, standardization, and reduced user dependence.

R2.23. Comment Discussion: Lines 261: "minimize general and systematic biases." A brief explanation of how you implemented this would be very helpful.

Response: We agree that the original statement would have required a more specific explanation of how such biases were assessed and minimized. Because this level of methodological detail was not systematically quantified in the present study, we removed this wording from the revised Discussion rather than leaving an insufficiently supported claim in the manuscript.

R2.24. Comment Methods: A statistical analysis section should be added with the full analysis (e.g., tests to check normality of the data), followed by Mann-Whitney previously mentioned in the figure legend and any other tests performed.

Response: Done. The Methods now include a dedicated statistical analysis subsection describing the unit of analysis, normality assessment, statistical tests, post hoc testing, and the use of image-level aggregation to avoid pseudoreplication. The figure legends showing our results (Fig 6 and 7) were revised accordingly. (Revised line 501-509)

R2.25. Comment Methods: There are several instances mentioning that "out-of-focus regions were excluded". I could not find a description on how this process was carried out. Could the authors expand "Training the deep learning network" with how this was done, how many operators and how it influences the results?

Response: We thank the reviewer for this important point. We have added this information to both the annotation/training description and the Results. In brief, out-of-focus regions were identified

manually during annotation based on predefined morphological criteria, including (i) an apparent widening of the tight-junction signal by more than 100% compared with adjacent in-focus regions and/or (ii) a clear straightening or loss of the characteristic meandering border appearance. Such regions, together with background noise and non-junctional signal, were annotated as negative class and were therefore explicitly included during training. This strategy was intended to reduce interference from blurred structures in downstream segmentation and morphometric analysis. Although the initial annotation step was manual, it followed standardized criteria and was cross-checked by two readers, so that user dependence was reduced as much as feasible within the annotation process. We also added further information about the whole training/annotation process. (Revised line 451-492)

R2.26. Comment Methods: Line 319: Can the authors add details regarding train-validation-test splits of the dataset and potential cross-validation evaluation of the performance? Additionally, I would expect a baseline method to be presented (e.g. thresholding, briefly mentioned in the text), or manual annotation or a state-of-the-art model (like CellPose). Can the authors add such a comparison?

Response: The train/validation/test setup is now described explicitly (Revised line 479-481). In addition, we added a benchmark against established tools (CellProfiler, Cellpose-SAM, Tissue Analyzer) to provide context for the present workflow (S1 Text, S9 Fig).

R2.27. Comment Methods: "Image annotation was conducted in 3D Slicer" it would also be valuable to clarify how many operators performed annotations, their experience level and how these annotations were homogenised to define the ground truth / training data.

Response: Done. The revised Methods now specify who performed annotation, how supervision was provided, and how disagreements were resolved. (Revised line 452-471)

R2.28. Comment Figures: Figure 4: can cell #18 be added to A for completeness, so this reference doesn't appear unexpectedly in C. Also unclear why it is numbered with 18 if no other cells are labelled in the figure. Perhaps it could just be "Example" or similar.

Response: Done. The figure labeling was simplified. (Revised Figure 5)

R2.29. Comment Figures: Figure 5: "Wildtype", typo? (or Wild-type to be consistent with other instances like in line 181). Can you provide a measure of dispersion in table C? (Since you are reporting means, the standard deviation?)

Response: Done. Terminology was corrected, dispersion measures were added, and the statistics were revised to include multiple-testing correction and effect size reporting. (Revised Figure 6)

R2.30. Comment Figures: Figure 6: There are visible instances in B of cells that appear merged (either due to lack of staining or segmentation artefacts). How are these handled?

Response: We agree with the reviewer that panel B shows instances of under-segmentation, where neighboring cells are erroneously merged. In the revised manuscript, we now explicitly state these erros and indicate the current limitations of direct model transfer to this dataset. We therefore revised the text to present this example as a proof-of-principle transfer rather than robust automated application, and we clarify that dataset-specific fine-tuning/retraining is required before reliable quantitative analysis can be performed. (Revised S8 Figure)

R2.31. Comment Figures: Figure S4: Light-green regions should also be labelled. It is also unclear how the small gray areas inside green regions are handled. In B, how are there contours for cells which are nearly invisible in A?

Response: We thank the reviewer for this helpful comment. We revised the figure legend to clarify the channel assignment and the genotype-classification procedure. Panel A shows the GFP channel only, whereas the cell contours in panel B were obtained from segmentation of the corresponding ZO-1 image and then overlaid onto the GFP signal, explaining why contours are visible even when GFP is weak. Details of the overlap-based genotype assignment (including handling of small GFP-negative regions within GFP-positive cells) are now described explicitly in the legend of S6 Fig.

R2.32. Comment Figures: Figure S5: Is panel A part of panel B? If yes, can it be indicated in B?

Response: Thanks for the suggestion, we have updated the figure accordingly. (Revised S7 Figure)

R2.33 Comment Data/code availability: The authors did a fantastic job with their documentation, and supporting screenshots. For the repository, I would consider adding the details regarding software requirements and versions already in the main Readme file.

Response: We appreciate the reviewer's kind remarks regarding the documentation. Following this suggestion, we updated the repository's main README to include the relevant software requirements and version information.

Reviewer 3

R3.1. Comment: "we present a fully automated, deep learning-based image analysis pipeline to quantify epithelial cell shape and tight junction morphology from immunofluorescence images." Details on the development and the features of the pipline are missing. How was the training performed? What kind of training images? Why were they only partly labeled? Are 9 images enough? Was there a validation and test data set?

Response: We thank the reviewer for highlighting this crucial aspect. We expanded the Methods substantially to address all of these points. The revised manuscript now describes the annotation strategy, the composition of the training/validation/test sets, the rationale for partial labeling of analyzable in-focus junction regions, and the fact that performance was assessed on an independent test set. We also clarify that the current training set size was sufficient for this narrowly defined assay, while broader transfer will require additional dataset-specific training. (Revised line 451-492)

R3.2. Comment: Regarding the measurements from the pipeline: How was the circularity calculated (since there are different possible definitions around)?

Response: We thank the reviewer for pointing out that the definition of circularity should be stated explicitly, as different formulations are used in the literature and across software platforms. In our workflow, circularity was calculated using the Mathematica ComponentMeasurements implementation as $2\pi r_{eq} / p$, where r_{eq} is the equivalent-disk radius of the segmented object and p its polygonal perimeter. This corresponds to the square root of the conventional circularity definition $4\pi A / P^2$. We have now added this information the Fig 4 legend.

R3.3. Comment: Can the authors comment on the neighbour count, considering that a lot of cells seem to be missing from the segmentation? Can they give a quantification of how many cells are discarded?

Response: We thank the reviewer for this helpful comment. Neighbor count was not determined by visually counting adjacent cells in the raw image, but was derived from the segmented and skeletonized ZO-1 network. Specifically, the junction network was separated at branch points, and the number of resulting border segments connected to each cell object was used to define neighbor count. We also added a manual review of a representative subset quantifying how many cells were excluded and for which reasons in the revised Results section. In this subset, 734 of 1,173 cells (62.6%) were excluded by the predefined quality-control criteria, including 38.9% due to border contact and 23.7% due to out-of-focus regions or segmentation errors, while 37.4% were retained for downstream analysis. Because additional biological replicates and image fields can be generated relatively easily in this system, we chose a deliberately conservative filtering strategy in order to avoid including incorrectly segmented cells in the downstream analysis. This was intended to prioritize detection quality over maximal cell yield. (Revised line 188-200)

R3.4. Comment: In line 137 it says that algorithms were standardized. What does this mean?

Response: We agree with the reviewer that the original statement "All algorithms were standardized" was too unspecific and could be misunderstood as implying phenotype-dependent normalization. We have therefore replaced this wording and now explicitly describe which fixed quality-control (QC) and exclusion steps were applied identically to all datasets and genotypes. Importantly, these steps do not normalize biological measurements (e.g., cell size, perimeter, or junctional morphology) within a dataset. Instead, they remove objects that cannot be reliably quantified due to technical artifacts (e.g., truncation at image borders, non-closed junction contours, merged-cell candidates, or defocus/segmentation failures). All remaining objects are subsequently quantified in physical units using the microscope pixel size, without dataset- or genotype-specific scaling, thereby preserving true biological differences. (Revised line 188-200)

R3.5. Comment: "employs a U-Net convolutional neural network for accurate segmentation of fluorescence labeled tight junctions" The analysis of segmentation accuracy in line 116-120 is not comprehensible. Details on how many researchers, the different background signals and artifacts, the number of images, the procedure for the calculation of the intersection over union, the details on the labeling strategy for the tight junctions, etc are missing.

Response: We expanded both the Results and Methods to provide more detail on the annotation strategy, image sets, evaluation metrics, number of reviewers involved in visual review, and the interpretation of the overlap metrics for thin junction structures. (Revised line 161-168, 451-492)

R3.6. Comment: "novel algorithms to quantify overall cell shape and tight junction morphology". A discussion of existing morphological measures in the introduction is missing, so that a reader can judge the novelty.

Response: We thank the reviewer for this important comment. We have revised the Introduction to better position our work relative to existing morphological measures. Specifically, we now discuss previously used approaches for epithelial morphology analysis, including manual quantification of tight-junction tortuosity, ruffling, and zig-zag morphology, as well as more general image-analysis tools for epithelial segmentation and morphometry. We now also clarify that such approaches have been informative, but are typically manual or semi-manual, user-dependent, and often restricted to relatively small numbers of selected junction segments rather than whole monolayers. (Revised line 95-103)

To better reflect this context, we revised the text to make clear that the novelty of our study does not lie in introducing epithelial morphology as a new biological concept, but in providing an automated and scalable workflow for assay-specific quantification of these phenotypes. In particular,

our approach is designed to preserve fine tight-junction contour irregularities and to extract descriptors tailored to this readout, including junction meandering and genotype-resolved analysis of shared borders in mixed-cell experiments. We hope this revision makes the methodological advance and its distinction from previous small-scale manual analyses clearer.

R3.7. Comment: Furthermore, the details of the R-index calculation are missing. What does smoothed mean? And how robust is the R-index regarding this smoothing procedure? would expect it to be highly dependent on the amount of smoothing.

Response: We thank the reviewer for this comment. The R-Index calculation is shown in Fig 5C. We clarified the meaning of smoothing procedure (combination of blurring, erosion and edge filters) and also added an additional Figure (S5 Fig) showing the effect on the whole image. We agree that the R-index depends on the strength of the smoothing, since it is based on a modeled representation of junctional contours. We therefore interpret it as a parameter-dependent readout within a standardized pipeline rather than as an absolute quantity. In the present study, the same smoothing settings were applied across all conditions, allowing relative differences between groups to be compared within a common analytical framework. We clarified this point in the revised Discussion. (Revised line 349-352)

R3.8. Comment: What are P, A, and C in Fig 4B?

Response: P, A, and C denote perimeter, area, and circularity, respectively. This information is stated in the figure legend. (Revised Fig 5)

R3.9. Comment: In Line 162, the authors mention a comparison with manual scoring of the R-index? What are the details? I am not sure how humans would score an R-index.

Response: We agree that the original wording was misleading. The revised text now describes manual assessment more carefully as a blinded qualitative reference for junctional morphology rather than as manual scoring of the numerical R-index itself.

R3.10. Comment: "Estimate cytoskeletal traction at shared cell borders" The section about cytoskeletal traction is not clear (line 176-185). What is calculated here, why is it calculated, and how is it done, and what is the conclusion from those calculations? And what is the motivation and purpose of the co-cultures in Fig S4?

Response: We thank the reviewer for this comment and agree that the original section was not sufficiently clear. We revised the manuscript to state explicitly what is computed, why it is computed, how it is computed, and what can be concluded, including a sample dataset (Fig 7).

We compute a border-level geometric proxy for junctional deformation from the segmented ZO-1 network to test whether the riffled/meandering phenotype is associated with altered mechanical coupling across shared cell–cell borders. After skeletonization, the junction graph is decomposed into border segments between neighboring cell pairs. For each segment, we quantify lateral deflection as the signed enclosed area between the segmented border polyline and the straight chord connecting the same endpoints (S6 Fig C). The chord is used only as a reference baseline; the enclosed area summarizes deviation from this baseline and corresponds to the integral of lateral displacement along the segment, enabling directional analysis at heterotypic borders.

Using WT.GFP–$\mathit{CGN}^{-/-}$ co-cultures, this framework yields a practical result that is difficult to obtain by visual inspection alone: the R-index differs systematically by border pairing (CNG–CNG < CNG–WT < WT–WT), and the enclosed-area proxy reveals a directional bias at WT–CNG borders (shift toward WT), whereas WT–WT borders show no bias (Fig 7C, D). The motivation for the co-culture

experiments is that they generate homotypic and heterotypic borders within the same epithelial field, allowing direct comparison under identical imaging conditions and directional assignment via GFP (Revised line 259-281, 356-368)

R3.11. Comment: "objectively identifies subtle morphogenetic changes associated with disease-related mutations, applied to a genetically modified Madin-Darby Canine Kidney cell model of ADPKD". Why do you call the morphological changes subtle? I would argue that for the human eye they are very obvious.

Response: We thank the reviewer for this important point. We agree that the original wording was imprecise, since genotype-associated differences are clearly visible in some representative images. We therefore revised the text to clarify that phenotypic strength varies across individual fields, with some images showing pronounced and others more moderate differences, as illustrated in the new S2 Fig. The manuscript now emphasizes that quantitative analysis is useful because it provides an objective measure of junctional meandering and allows effect sizes to be compared across genotypes and experiments, rather than implying that the phenotype is uniformly subtle to the human eye.

R3.12. Comment: And more importantly, it was not clear why it is important to measure these differences and which biomedical conclusions are drawn from these measurements.

Response: We thank the reviewer for this important point. We revised the Introduction to better motivate why quantifying tight-junction "riffled/meandering" patterns is biologically and biomedically relevant and what can be concluded from these measurements. Specifically, similar junctional meandering/ruffling phenotypes have been reported across multiple biological settings, suggesting that they reflect conserved changes in epithelial organization and mechanical coupling at cell–cell contacts; we therefore added the relevant literature to the Introduction. However, prior work has largely relied on qualitative description or low-throughput, manually curated measurements, making standardized comparisons across genotypes, conditions, and experiments difficult. The quantitative readouts introduced here, tight-junction configuration metrics and the border-level deformation (traction-proxy) measure, address this gap by enabling scalable, standardized detection and comparison of genotype-associated differences in junctional architecture and force transmission, including subtle phenotypes that are not reliably captured by blinded visual inspection alone.

R3.13. Comment: Also, in Fig 5 you should apply a correction for multiple testing and present an effect size.

Response: Done. The statistics were revised accordingly. (Revised Fig 6)

R3.14. Comment: Additionally, it would be interesting to determine the level of difference in R-index that one would consider biomedically relevant.

Response: We thank the reviewer for this helpful comment. We agree that defining a biomedically relevant threshold for R-index differences would be valuable. However, we do not believe that such a threshold can be derived reliably and depend on the specific experimental context. This was added in the Discussion. (Revised line 349-352)

R3.15. Comment: „applying it to Drosophila egg chamber epithelia" The segmentation result for a single image is shown. This should be accompanied by a quantification of the segmentation performance and ideally extended to more images that can certainly be obtained from other publications. In this example the R-index does not seem to be relevant.

Response: We agree that the evidence shown in the original figure was limited and that a single example image is insufficient to support a broader claim of general applicability. We therefore revised the manuscript to present this figure as an illustrative transfer example rather than as formal performance validation. In addition, we removed overly strong wording in both the main text and the figure legend. We also agree that the R-index is not informative in this context and have revised the presentation accordingly. The figure legend now explicitly highlights that the shown output includes under-segmentation/merged cells, illustrating the current limitations of direct transfer without dataset-specific retraining.

R3.16. Comment: "broad applications in phenotypic screening, disease modeling, and morphogenesis research" The broad applicability of the pipeline is not clear to me. I agree that a segmentation pipeline for epithelia with cell outline staining is of great interest. But it did not become clear why the presented tool is relevant, considering there are a number of existing ones, such as Cellpose, Tissue Analyser, or others. As mentioned above, for the two examples given, the applicability of the the R-index did not become clear.

Response: We thank the reviewer for this comment. We agree that the original wording overstated the breadth of applicability supported by the present study. We therefore removed the broad claim and revised the manuscript to define the method more narrowly. At the same time, we now clarify that the observed junctional phenotype is of broader biological interest, but has so far mainly been assessed manually in relatively small sample sets. Automated quantification of such phenotypes may therefore be useful beyond the present model system. The revised Discussion also makes clearer that the contribution of the workflow is not generic epithelial segmentation, but assay-specific quantification of junctional meandering and shared-border behavior in mixed-cell experiments, which is not directly addressed by existing general-purpose morphology tools (see benchmarking in S1 Text, S9 Fig). Finally, we now present the R-index as a context-dependent readout for the systems analyzed here, rather than as a broadly validated general-purpose metric.

R3.17. Comment: Fig 5C: there should be decimal dots instead of commas

Response: Done.

R3.18. Comment: References of subpanels of Fig S4 in the text are in the wrong order

Response: Done. The text was corrected during revision.

R3.19. Comment: Methods: a cell line *CGN*$^{-/-}$ is mentioned. Which results correspond to this cell line?

Response: We clarified this in the revised manuscript by linking the *CGN*$^{-/-}$ line explicitly to the co-culture experiments and the related results. (Revised 270-281, Fig 7)

Journal / technical comments

J1. Comment: We have noticed that you have uploaded Supporting Information files, but you have not included a list of legends. Please add a full list of legends for your Supporting Information files after the references list.

Response: We added the list of legends.

J2. Comment: Potential copyright issue regarding Fig. S3D / map base layer.

Response: This item is not applicable. The base layer used for the figure is our own immunofluorescence image (S3A; S4A in new numbering) rather than an external map source.

J3. Comment: Please update the data availability statement and deposit the minimal dataset.

Response: The raw immunofluorescence data and analysis outputs have been organized for public availability, and archival deposition via Zenodo has been prepared/updated (10.5281/zenodo.15857671) alongside the code repository.

J4. Comment: Data availability.

Response: Source code and all data shown in the manuscript is available on GitHub at https://github.com/JahnJ/U-Net-based-image-quantification-of-epithelial-cell-shapes/. We have also used Zenodo to assign a DOI to the repository: 10.5281/zenodo.15857671.

J5. Comment: Please amend the financial disclosure statement.

Response: M.K. was funded by the German Research Foundation (DFG) project ID 431984000 (SFB 1453). and project ID 239283807 (TRR 152). A-K.C. and M.K. were supported by Germany's Excellence Strategy (CIBSS, EXC-2189, project ID 390939984). Lighthouse Core Facility is funded in part by the Medical Faculty, University of Freiburg (Project Numbers 2023/A2-Fol; 2021/B3-Fol) the DTK and the DFG (Project Number 450392965). The funders had no role in study design, data collection and analysis, decision to publish, or preparation of the manuscript.

This information was also added in the manuscript.
