# Supplementary material for: Deep learning-supported image quantification of epithelial cell shapes and its application to polycystic kidney disease
Source: PLoS Comput Biol. 2026 Aug 3;22(8):e1014614. doi: 10.1371/journal.pcbi.1014614 (PMC13456487; doi:10.1371/journal.pcbi.1014614)
Supplement: Supplementary file 6 [file pcbi.1014614.s017.pdf]

Review of the paper for PLOS

Deep learning-supported image quantification of epithelial cell shapes

Comments about the reviews and paper improvements for PLOS

Thanks to the authors for addressing all my concerns and putting in the effort to improve the paper.

Reply to Comment R1.1.1: I agree with the new wording on the new paragraph regarding as it’s more specific and accurate.

Reply to Comment R1.1.2 and Comment R1.13: I appreciate the adjustment of the statements and the benchmark on the supplementary helps to clarify the positions of the author’s workflow VS the state of art.

Reply to Comment R1.1.3: Acknowledgement that authors addressed the comment and decided to keep the title.

Reply to Comment R1.3: The new reward helps to understand the other tools better in the context of the paper.

Reply to Comment R1.1.4, Comment R1.2, Comment R1.4, Comment R1.5, Comment R1.6, Comment R1.7, Comment R1.8, Comment R1.9, Comment R1.10, Comment R1.11, Comment R1.12: Previous iterations acknowledgements

Reply Comment R1.5, Comment R1.14: I understand that the reimplementation is out of the scope and thank the authors for acknowledging that it may be a future step to take for next iterations not to that paper.

I thank the authors for acknowledging all the observations and comments and I’d like to congratulate them for their efforts and their good work.
