# Supplementary material for: Deep learning-supported image quantification of epithelial cell shapes and its application to polycystic kidney disease
Source: PLoS Comput Biol. 2026 Aug 3;22(8):e1014614. doi: 10.1371/journal.pcbi.1014614 (PMC13456487; doi:10.1371/journal.pcbi.1014614)
Supplement: Peer review history

# Peer review: Decision Letter 0

**DOI:** 10.1371/journal.pcbi.1014614.r001

**Authors:** Boyce Griffith, Virginie Uhlmann

17 Feb 2026

Deep learning-based image quantification of epithelial cell shapes and its application to polycystic kidney disease

PLOS Computational Biology

Dear Dr. Kottgen,

Thank you for submitting your manuscript to PLOS Computational Biology. After careful consideration, we feel that it has merit but does not fully meet PLOS Computational Biology's publication criteria as it currently stands. Therefore, we invite you to submit a revised version of the manuscript that addresses the points raised during the review process.

Please submit your revised manuscript by Apr 19 2026 11:59PM. If you will need more time than this to complete your revisions, please reply to this message or contact the journal office at ploscompbiol@plos.org. When you're ready to submit your revision, log on to https://www.editorialmanager.com/pcompbiol/ and select the 'Submissions Needing Revision' folder to locate your manuscript file.

Please include the following items when submitting your revised manuscript:

* A rebuttal letter that responds to each point raised by the editor and reviewer(s). You should upload this letter as a separate file labeled 'Response to Reviewers'. This file does not need to include responses to formatting updates and technical items listed in the 'Journal Requirements' section below.

* A marked-up copy of your manuscript that highlights changes made to the original version. You should upload this as a separate file labeled 'Revised Manuscript with Track Changes'.

* An unmarked version of your revised paper without tracked changes. You should upload this as a separate file labeled 'Manuscript'.

If you would like to make changes to your financial disclosure, competing interests statement, or data availability statement, please make these updates within the submission form at the time of resubmission. Guidelines for resubmitting your figure files are available below the reviewer comments at the end of this letter

We look forward to receiving your revised manuscript.

Kind regards,

Virginie Uhlmann

Academic Editor

PLOS Computational Biology

Boyce Griffith

Section Editor

PLOS Computational Biology

**Journal Requirements:**

If the reviewer comments include a recommendation to cite specific previously published works, please review and evaluate these publications to determine whether they are relevant and should be cited. There is no requirement to cite these works unless the editor has indicated otherwise.

1) We ask that a manuscript source file is provided at Revision. Please upload your manuscript file as a .doc, .docx, .rtf or .tex. If you are providing a .tex file, please upload it under the item type u2018LaTeX Source Fileu2019 and leave your .pdf version as the item type u2018Manuscriptu2019.

2) Please upload all main figures as separate Figure files in .tif or .eps format. For more information about how to convert and format your figure files please see our guidelines:

https://journals.plos.org/ploscompbiol/s/figures

3) We have noticed that you have uploaded Supporting Information files, but you have not included a list of legends. Please add a full list of legends for your Supporting Information files after the references list.

4) Some material included in your submission may be copyrighted. According to PLOSu2019s copyright policy, authors who use figures or other material (e.g., graphics, clipart, maps) from another author or copyright holder must demonstrate or obtain permission to publish this material under the Creative Commons Attribution 4.0 International (CC BY 4.0) License used by PLOS journals. Please closely review the details of PLOSu2019s copyright requirements here: PLOS Licenses and Copyright. If you need to request permissions from a copyright holder, you may use PLOS's Copyright Content Permission form.

Please respond directly to this email and provide any known details concerning your material's license terms and permissions required for reuse, even if you have not yet obtained copyright permissions or are unsure of your material's copyright compatibility. Once you have responded and addressed all other outstanding technical requirements, you may resubmit your manuscript within Editorial Manager.

Potential Copyright Issues:

i) Figure S3D. Please (a) provide a direct link to the base layer of the map (i.e., the country or region border shape) and ensure this is also included in the figure legend; and (b) provide a link to the terms of use / license information for the base layer image or shapefile. We cannot publish proprietary or copyrighted maps (e.g. Google Maps, Mapquest) and the terms of use for your map base layer must be compatible with our CC BY 4.0 license.

Note: if you created the map in a software program like R or ArcGIS, please locate and indicate the source of the basemap shapefile onto which data has been plotted.

If your map was obtained from a copyrighted source please amend the figure so that the base map used is from an openly available source. Alternatively, please provide explicit written permission from the copyright holder granting you the right to publish the material under our CC BY 4.0 license.

If you are unsure whether you can use a map or not, please do reach out and we will be able to help you. The following websites are good examples of where you can source open access or public domain maps:

* U.S. Geological Survey (USGS) - All maps are in the public domain. (http://www.usgs.gov)

* PlaniGlobe - All maps are published under a Creative Commons license so please cite u201cPlaniGlobe, http://www.planiglobe.com, CC BY 2.0u201d in the image credit after the caption. (http://www.planiglobe.com/?lang=enl)

* Natural Earth - All maps are public domain. (http://www.naturalearthdata.com/about/terms-of-use/).

5) In the online submission form, you indicated that your data will be submitted to a repository upon acceptance. We strongly recommend all authors deposit their data before acceptance, as the process can be lengthy and hold up publication timelines. Please note that, though access restrictions are acceptable now, your entire minimal dataset will need to be made freely accessible if your manuscript is accepted for publication. This policy applies to all data except where public deposition would breach compliance with the protocol approved by your research ethics board. If you are unable to adhere to our open data policy, please kindly revise your statement to explain your reasoning and we will seek the editor's input on an exemption.

6) Please amend your detailed Financial Disclosure statement. This is published with the article. It must therefore be completed in full sentences and contain the exact wording you wish to be published.

1) State the initials, alongside each funding source, of each author to receive each grant. For example: "This work was supported by the National Institutes of Health (####### to AM; ###### to CJ) and the National Science Foundation (###### to AM)."

2) State what role the funders took in the study. If the funders had no role in your study, please state: "The funders had no role in study design, data collection and analysis, decision to publish, or preparation of the manuscript."

3) If any authors received a salary from any of your funders, please state which authors and which funders..

If you did not receive any funding for this study, please simply state: u201cThe authors received no specific funding for this work.u201d

**Reviewers' comments:**

Reviewer's Responses to Questions

**Comments to the Authors:**

**Please note here if the review is uploaded as an attachment.**

Reviewer #1: My review is in the attach as a PDF. Thank you for your work.

Reviewer #2: The manuscript explores the use of deep-learning (DL) models to support the segmentation of epithelial cell contours from microscopy images with immunofluorescence staining and quantify their morphometry. Deep learning models for image analysis tasks have matured considerably over the last years, making established models promising for challenging segmentation tasks, such as the segmentation of cell contours presented here. This is a valuable application area where automation could significantly benefit the field. While the application of deep learning models is potentially valuable, I believe the manuscript requires improvements in problem contextualization within existing automated approaches, methodology description and comparison, and statistical rigor. Please find some comments attached.

Reviewer #3: Jahn et al. present an image analysis pipeline that quantifies cell morphology from two-dimensional fluorescent images with cell outline staining. It is an interesting idea and may have relevance in some fields, but the pipeline is described only superficially, its performance is not rigorously evaluated, and the biological relevance is not clearly described in the manuscript.

I’ve organised my review according to the claims in the abstract:

“we present a fully automated, deep learning-based image analysis pipeline to quantify epithelial cell shape and tight junction morphology from immunofluorescence images.“

Details on the development and the features of the pipline are missing. How was the training performed? What kind of training images? Why were they only partly labeled? Are 9 images enough? Was there a validation and test data set? Regarding the measurements from the pipeline: How was the circularity calculated (since there are different possible definitions around)? Can the authors comment on the neighbour count, considering that a lot of cells seem to be missing from the segmentation? Can they give a quantification of how many cells are discarded? In line 137 it says that algorithms were standardized. What does this mean?

“employs a U-Net convolutional neural network for accurate segmentation of fluorescence labeled tight junctions”

The analysis of segmentation accuracy in line 116-120 is not comprehensible. Details on how many researchers, the different background signals and artifacts, the number of images, the procedure for the calculation of the intersection over union, the details on the labeling strategy for the tight junctions, etc are missing.

“novel algorithms to quantify overall cell shape and tight junction morphologynovel algorithms to quantify overall cell shape and tight junction morphology”

A discussion of existing morphological measures in the introduction is missing, so that a reader can judge the novelty. Furthermore, the details of the R-index calculation are missing. What does smoothed mean? And how robust is the R-index regarding this smoothing procedure? I would expect it to be highly dependent on the amount of smoothing. What are P, A, and C in Fig 4B? In Line 162, the authors mention a comparison with manual scoring of the R-index? What are the details? I am not sure how humans would score an R-index.

“Estimate cytoskeletal traction at shared cell borders”

The section about cytoskeletal traction is not clear (line 176-185). What is calculated here, why is it calculated, and how is it done, and what is the conclusion from those calculations? And what is the motivation and purpose of the co-cultures in Fig S4?

“objectively identifies subtle morphogenetic changes associated with disease-related mutations, applied to a genetically modified Madin-Darby Canine Kidney cell model of ADPKD”

Why do you call the morphological changes subtle? I would argue that for the human eye they are very obvious. And more importantly, it was not clear why it is important to measure these differences and which biomedical conclusions are drawn from these measurements. Also, in Fig 5 you should apply a correction for multiple testing and present an effect size. Additionally, it would be interesting to determine the level of difference in R-index that one would consider biomedically relevant.

“applying it to Drosophila egg chamber epithelia”

The segmentation result for a single image is shown. This should be accompanied by a quantification of the segmentation performance and ideally extended to more images that can certainly be obtained from other publications. In this example the R-index does not seem to be relevant.

“broad applications in phenotypic screening, disease modeling, and morphogenesis research”

The broad applicability of the pipeline is not clear to me. I agree that a segmentation pipeline for epithelia with cell outline staining is of great interest. But it did not become clear why the presented tool is relevant, considering there are a number of existing ones, such as Cellpose, Tissue Analyser, or others. As mentioned above, for the two examples given, the applicability of the the R-index did not become clear.

Minor comments:

Fig 5C: there should be decimal dots instead of commas

References of subpanels of Fig S4 in the text are in the wrong order

Methods: a cell line Cgn-/- is mentioned. Which results correspond to this cell line?

**********

**Have the authors made all data and (if applicable) computational code underlying the findings in their manuscript fully available?**

The PLOS Data policy requires authors to make all data and code underlying the findings described in their manuscript fully available without restriction, with rare exception (please refer to the Data Availability Statement in the manuscript PDF file). The data and code should be provided as part of the manuscript or its supporting information, or deposited to a public repository. For example, in addition to summary statistics, the data points behind means, medians and variance measures should be available. If there are restrictions on publicly sharing data or code —e.g. participant privacy or use of data from a third party—those must be specified.

Reviewer #1: Yes

Reviewer #2: Yes

Reviewer #3: Yes

**********

PLOS authors have the option to publish the peer review history of their article (what does this mean?). If published, this will include your full peer review and any attached files.

If you choose “no”, your identity will remain anonymous but your review may still be made public.

**Do you want your identity to be public for this peer review?**  For information about this choice, including consent withdrawal, please see our Privacy Policy.

Reviewer #1: No

Reviewer #2: No

Reviewer #3: No

[NOTE: If reviewer comments were submitted as an attachment file, they will be attached to this email and accessible via the submission site. Please log into your account, locate the manuscript record, and check for the action link "View Attachments". If this link does not appear, there are no attachment files.]

**Figure resubmission:**

While revising your submission, we strongly recommend that you use PLOS’s NAAS tool (https://ngplosjournals.pagemajik.ai/artanalysis) to test your figure files. NAAS can convert your figure files to the TIFF file type and meet basic requirements (such as print size, resolution), or provide you with a report on issues that do not meet our requirements and that NAAS cannot fix.

After uploading your figures to PLOS’s NAAS tool - https://ngplosjournals.pagemajik.ai/artanalysis, NAAS will process the files provided and display the results in the "Uploaded Files" section of the page as the processing is complete. If the uploaded figures meet our requirements (or NAAS is able to fix the files to meet our requirements), the figure will be marked as "fixed" above. If NAAS is unable to fix the files, a red "failed" label will appear above. When NAAS has confirmed that the figure files meet our requirements, please download the file via the download option, and include these NAAS processed figure files when submitting your revised manuscript.

**Reproducibility:**

To enhance the reproducibility of your results, we recommend that authors of applicable studies deposit laboratory protocols in protocols.io, where a protocol can be assigned its own identifier (DOI) such that it can be cited independently in the future. Additionally, PLOS ONE offers an option to publish peer-reviewed clinical study protocols. Read more information on sharing protocols at https://plos.org/protocols?utm_medium=editorial-email&utm_source=authorletters&utm_campaign=protocols

Attachment Submitted filename: 20251106_reviewsPLOSKidneyDL.pdf

Attachment Submitted filename: ManuscriptComments.pdf

# Author comment: Author response to Decision Letter 1

**DOI:** 10.1371/journal.pcbi.1014614.r002

30 Mar 2026

Attachment Submitted filename: R1 Response to Reviewers.pdf

# Peer review: Decision Letter 1

**DOI:** 10.1371/journal.pcbi.1014614.r003

**Authors:** Boyce Griffith, Virginie Uhlmann

12 May 2026

Deep learning-supported image quantification of epithelial cell shapes and its application to polycystic kidney disease

PLOS Computational Biology

Dear Dr. Kottgen,

Thank you for submitting your manuscript to PLOS Computational Biology. After careful consideration, we feel that it has merit but does not fully meet PLOS Computational Biology's publication criteria as it currently stands. Therefore, we invite you to submit a revised version of the manuscript that addresses the points raised during the review process.

Please submit your revised manuscript by Jul 12 2026 11:59PM. If you will need more time than this to complete your revisions, please reply to this message or contact the journal office at ploscompbiol@plos.org. When you're ready to submit your revision, log on to https://www.editorialmanager.com/pcompbiol/ and select the 'Submissions Needing Revision' folder to locate your manuscript file.

Please include the following items when submitting your revised manuscript:

* A letter that responds to each point raised by the editor and reviewer(s). You should upload this letter as a separate file labeled 'Response to Reviewers'. This file does not need to include responses to formatting updates and technical items listed in the 'Journal Requirements' section below.

* A marked-up copy of your manuscript that highlights changes made to the original version. You should upload this as a separate file labeled 'Revised Manuscript with Track Changes'.

* An unmarked version of your revised paper without tracked changes. You should upload this as a separate file labeled 'Manuscript'.

We look forward to receiving your revised manuscript.

Kind regards,

Virginie Uhlmann

Academic Editor

PLOS Computational Biology

Boyce Griffith

Section Editor

PLOS Computational Biology

**Additional Editor Comments (if provided):**

**Journal Requirements:**

If the reviewer comments include a recommendation to cite specific previously published works, please review and evaluate these publications to determine whether they are relevant and should be cited. There is no requirement to cite these works unless the editor has indicated otherwise.

[Note: HTML markup is below. Please do not edit.]

**Reviewers' comments:**

Reviewer's Responses to Questions

**Comments to the Authors:**

**Please note here if the review is uploaded as an attachment.**

Reviewer #1: I've attached a PDF file with my comments. Great effort and thank you for reply to all the points I made.

Reviewer #2: Thank you for the extensive replies to the comments raised previously, which have greatly improved my understanding of the work. I would have some additional minor topics to be clarified (please see PDF attached).

Reviewer #3: Thank you for your revised manuscript, which made several things clearer.

General comments:

Essentially, your requirement is to segment in-focus cell junctions while discarding out-of-focus ones. All the existing tools are developed for the segmentation of cells rather than tight junctions and therefore are developed to consider out of focus areas. Making this more explicit in the abstract and introduction would make the purpose of your work clearer. Since you later on also look at cell-specific measurements, this requirement gets lost in the remaining text. Streamlining your text with a clear focus would improve its readability.

The separation of the cell perimeter in the different branches of the tight junction network is a central point of your analysis. It should be illustrated in one of the figures.

Specific comments:

Line 177 “continued visual inspection over >2 years of workflow.” What is meant by that?

Line 196 “typical MSCK WT area …” How was this area measured? If you used the same pipeline for the measurement of the cell area, it would not be a valid argument.

Line 197 “subset of 12 images, … 1,173 cells” and Line 203 “5,477” Where these different subsets? How were they chosen? Why did you need different ones?

Line 241 – 245 provide more details on the classification test. How many images? Ideally, show a confusion matrix.

Line 272-274 You claim that you can use an area as an approximation for a force. Why is that valid? What are the assumptions that you have to make?

Legend of Fig 7 (B) “signed enclosed area” How do you decide on the sign of the area?

Line 318 -328, I do not think that you can make a conclusion about the performance of your pipeline for the Drosophila egg chamber from this one image. Undersegmentation also occurs for your own data set. You would need a systematic analysis of the percentage of undersegmentation in Drosophila. From my point of view, all you can state is that it is technically possible to feed this image into your pipeline.

**********

**Have the authors made all data and (if applicable) computational code underlying the findings in their manuscript fully available?**

The PLOS Data policy requires authors to make all data and code underlying the findings described in their manuscript fully available without restriction, with rare exception (please refer to the Data Availability Statement in the manuscript PDF file). The data and code should be provided as part of the manuscript or its supporting information, or deposited to a public repository. For example, in addition to summary statistics, the data points behind means, medians and variance measures should be available. If there are restrictions on publicly sharing data or code —e.g. participant privacy or use of data from a third party—those must be specified.

Reviewer #1: Yes

Reviewer #2: Yes

Reviewer #3: Yes

**********

PLOS authors have the option to publish the peer review history of their article (what does this mean?). If published, this will include your full peer review and any attached files.

If you choose “no”, your identity will remain anonymous but your review may still be made public.

**Do you want your identity to be public for this peer review?**  For information about this choice, including consent withdrawal, please see our Privacy Policy.

Reviewer #1: No

Reviewer #2: No

Reviewer #3: No

[NOTE: If reviewer comments were submitted as an attachment file, they will be attached to this email and accessible via the submission site. Please log into your account, locate the manuscript record, and check for the action link "View Attachments". If this link does not appear, there are no attachment files.]

**Figure resubmission:**

**Reproducibility:**

?>

Attachment Submitted filename: ManuscriptComments R1.pdf

Attachment Submitted filename: 20260420_commentsreviewsPLOSKidneyDL.pdf

# Author comment: Author response to Decision Letter 2

**DOI:** 10.1371/journal.pcbi.1014614.r004

28 May 2026

Attachment Submitted filename: R2 Response to Reviewers.docx

# Peer review: Decision Letter 2

**DOI:** 10.1371/journal.pcbi.1014614.r005

**Authors:** Boyce Griffith, Virginie Uhlmann

20 Jul 2026

Dear Dr. Kottgen,

We are pleased to inform you that your manuscript 'Deep learning-supported image quantification of epithelial cell shapes and its application to polycystic kidney disease' has been provisionally accepted for publication in PLOS Computational Biology.

Before your manuscript can be formally accepted you will need to complete some formatting changes, which you will receive in a follow up email. A member of our team will be in touch with a set of requests.

Please note that your manuscript will not be scheduled for publication until you have made the required changes, so a swift response is appreciated.

IMPORTANT: The editorial review process is now complete. PLOS will only permit corrections to spelling, formatting or significant scientific errors from this point onwards. Requests for major changes, or any which affect the scientific understanding of your work, will cause delays to the publication date of your manuscript.

Should you, your institution's press office or the journal office choose to press release your paper, you will automatically be opted out of early publication. We ask that you notify us now if you or your institution is planning to press release the article. All press must be co-ordinated with PLOS.

Thank you again for supporting Open Access publishing; we are looking forward to publishing your work in PLOS Computational Biology.

Best regards,

Virginie Uhlmann

Academic Editor

PLOS Computational Biology

Boyce Griffith

Section Editor

PLOS Computational Biology

***********************************************************

Reviewer's Responses to Questions

**Comments to the Authors:**

**Please note here if the review is uploaded as an attachment.**

Reviewer #1: I attached a pdf with my acknowledgement however, I don't have further comments.

Reviewer #2: Thank you for your comprehensive revisions and great work!

Reviewer #3: Thank you for this second review. I have no further comments.

**********

**Have the authors made all data and (if applicable) computational code underlying the findings in their manuscript fully available?**

The PLOS Data policy requires authors to make all data and code underlying the findings described in their manuscript fully available without restriction, with rare exception (please refer to the Data Availability Statement in the manuscript PDF file). The data and code should be provided as part of the manuscript or its supporting information, or deposited to a public repository. For example, in addition to summary statistics, the data points behind means, medians and variance measures should be available. If there are restrictions on publicly sharing data or code —e.g. participant privacy or use of data from a third party—those must be specified.

Reviewer #1: Yes

Reviewer #2: Yes

Reviewer #3: None

**********

PLOS authors have the option to publish the peer review history of their article (what does this mean?). If published, this will include your full peer review and any attached files.

If you choose “no”, your identity will remain anonymous but your review may still be made public.

**Do you want your identity to be public for this peer review?**  For information about this choice, including consent withdrawal, please see our Privacy Policy.

Reviewer #1: No

Reviewer #2: No

Reviewer #3: No

Attachment Submitted filename: 20260707_resubmitPLOSKidneyDL.pdf

# Editor report: Acceptance letter

**DOI:** 10.1371/journal.pcbi.1014614.r006

**Authors:** Boyce Griffith, Virginie Uhlmann

PCOMPBIOL-D-25-01691R2

Deep learning-supported image quantification of epithelial cell shapes and its application to polycystic kidney disease

Dear Dr Kottgen,

I am pleased to inform you that your manuscript has been formally accepted for publication in PLOS Computational Biology. Your manuscript is now with our production department and you will be notified of the publication date in due course.

The corresponding author will soon be receiving a typeset proof for review, to ensure errors have not been introduced during production. Please review the PDF proof of your manuscript carefully, as this is the last chance to correct any errors. Please note that major changes, or those which affect the scientific understanding of the work, will likely cause delays to the publication date of your manuscript.

Soon after your final files are uploaded, unless you have opted out, the early version of your manuscript will be published online. The date of the early version will be your article's publication date. The final article will be published to the same URL, and all versions of the paper will be accessible to readers.

For Research, Software, and Methods articles, you will receive an invoice from PLOS for your publication fee after your manuscript has reached the completed accept phase. If you receive an email requesting payment before acceptance or for any other service, this may be a phishing scheme. Learn how to identify phishing emails and protect your accounts at https://explore.plos.org/phishing.

Thank you again for supporting PLOS Computational Biology and open-access publishing. We are looking forward to publishing your work!

With kind regards,

Anitha Samidurai

PLOS Computational Biology | Carlyle House, Carlyle Road, Cambridge CB4 3DN | United Kingdom ploscompbiol@plos.org | Phone +44 (0) 1223-442824 | ploscompbiol.org | @PLOSCompBiol
